# Remodeling macrophage efferocytosis and phenotype *via* targeted nanoimmunotherapy for atherosclerosis

**DOI:** 10.1016/j.apsb.2026.08.006

**Published:** 2026-08-09

**Authors:** Zhongshan He, Yaoyao Luo, Yongjiang Li, Shuping Yang, Yingjun Fan, Yi Zhang, Shengbin Liu, Zhuoming Zhou, Chengjian Cao, Haixing Shi, Mengran Guo, Yuting Chen, Yupei Zhang, Duotian Qin, Wei Chen, Xiangrong Song

**Affiliations:** a Department of Critical Care Medicine, Department of Clinical Pharmacy, Frontiers Science Center for Disease-Related Molecular Network, State Key Laboratory of Biotherapy and Cancer Center, West China Hospital, Sichuan University, Tianfu Jincheng Laboratory, Chengdu 610041, China; b Laboratory of Cardiac Structure and Function, Institute of Cardiovascular Diseases, West China Hospital, Sichuan University, Chengdu 610041, China; c Center for Nanomedicine and Department of Anesthesiology, Brigham and Women's Hospital, Harvard Medical School, Boston, MA 02115, USA; d Department of Bioengineering, School of Engineering and Applied Science, University of Pennsylvania, Philadelphia, PA 19104, USA

**Keywords:** Macrophages, Efferocytosis, M2-like phenotype, Precise delivery, Nanotherapeutics, Plaque stability, Inflammation resolution, Atherosclerosis

## Abstract

Macrophages within advanced atherosclerotic lesions typically exhibit impaired efferocytosis and tend to adopt a pro-inflammatory M1-like phenotype, leading to the progression of atherosclerosis. Despite this challenge, the development of biocompatible nanotherapeutics capable of effectively remodeling macrophage efferocytosis and phenotype remains elusive. Here, we developed engineered targeted-peptide-modified nanocarriers (PP) and demonstrated their potential to restore macrophage efferocytosis while promoting re-polarization towards an anti-inflammatory M2-like phenotype. Furthermore, PP efficiently facilitated the delivery of an inflammation-resolving-drug to lesional macrophages within advanced atherosclerotic lesions. This combination approach substantially enhanced the anti-atherosclerotic efficacy of PP-based nanomedicine, effectively ameliorating atherosclerosis progression. In advanced plaque-bearing *ApoE*^−/−^ mice, PP nanomedcine efficiently accumulated in atherosclerotic plaques, notably restoring macrophage efferocytosis, promoting anti-inflammatory M2-like macrophage polarization, and leading to reduced plaque areas and enhanced plaque stability. This study not only underscores the new pleiotropic effects of PP to restore macrophage efferocytosis and modulate macrophage phenotype, but also highlights the promising potential of PP for the treatment of atherosclerosis and diseases associated with macrophage dysfunction.

## Graphical abstract

This study developed a targeted peptide-modified phosphorene nanosheet nanoplatform for treating atherosclerosis by restoring efferocytosis and promoting M2-like polarization of lesional macrophages, with potential for diseases associated with impaired efferocytosis.

**Figure:**
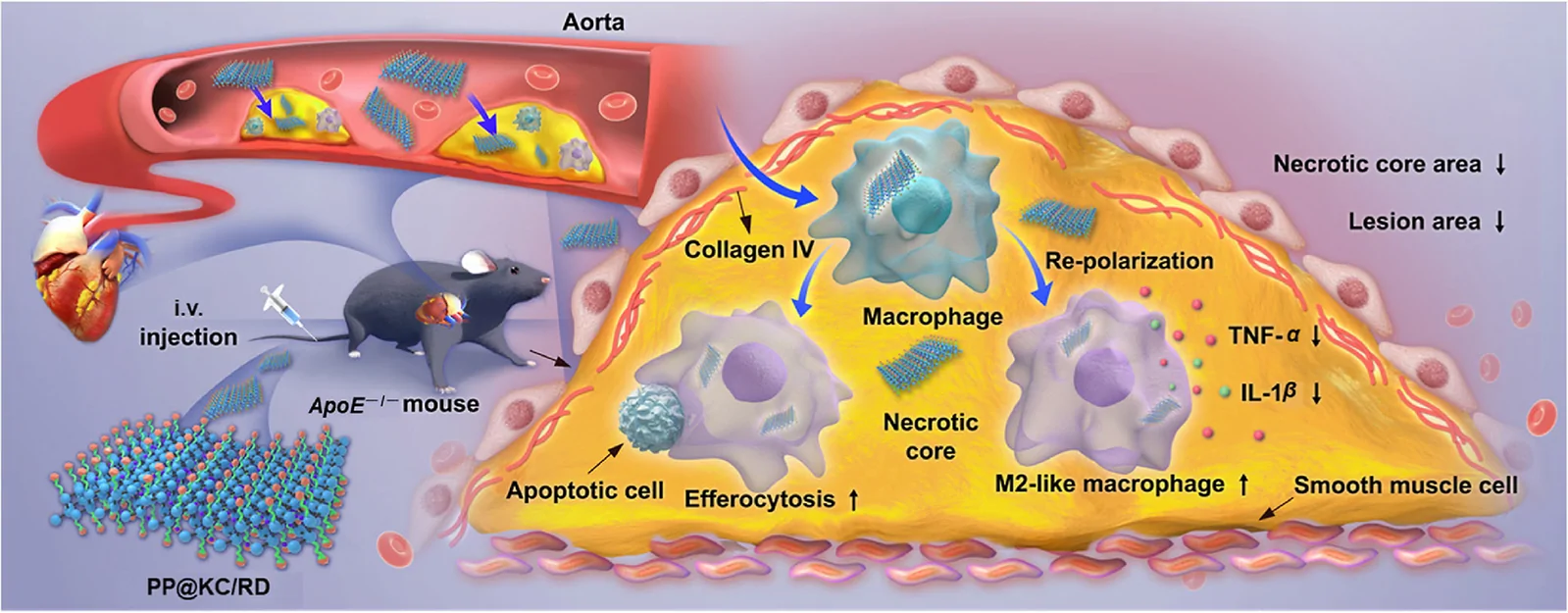

## 1 Introduction

Macrophages constitute the main immune cell population within atherosclerotic plaques and play pivotal roles in driving the progression of atherosclerosis^1^, ^2^, ^3^. Specifically, in advanced atherosclerotic plaques, lesional macrophages often exhibit impaired efferocytosis, leading to necrotic core enlargement and adverse plaque deposition, which can result in atherothrombotic events^4^, ^5^, ^6^. Consequently, restoring the efferocytic capacity of lesional macrophages to enhance the clearance of apoptotic cells holds therapeutic potentials in stabilizing vulnerable plaques and mitigating inflammatory responses^7^, ^8^, ^9^. Recent preclinical studies have shown that systemically administering an anti-CD47 antibody to atherosclerotic mice efficiently restored efferocytosis by macrophages^10^. Additionally, a first-in-human trial revealed that anti-CD47 antibody therapy successfully restored macrophage efferocytosis. While promising, this approach can lead to off-target clearance of healthy tissues and removal of red blood cells from the spleen, resulting in toxicities such as anemia^11^. Consequently, the effective targeted delivery of therapeutics to lesional macrophages that can restore the macrophage efferocytic capacity while mitigating off-target adverse effects emerges as a promising therapeutic strategy for atherosclerosis.

The polarization of pro-inflammatory M1-like macrophages within advanced atherosclerotic lesions is a critical contributor to the exacerbation of atherosclerotic progression. Conversely, the pro-resolving M2-like macrophages efficiently resolve inflammation and contribute to the regression of atherosclerotic lesions^12^, ^13^, ^14^. Notably, macrophage-mediated efferocytosis has the capability to induce polarization of the M2-like phenotype by increasing the production of specialized pro-resolving mediators, such as transforming growth factor-*β* (TGF-*β*) and interleukin-10 (IL-10)^15^. Therefore, developing a therapeutic strategy that simultaneously promotes M2-like phenotype polarization and restores macrophage efferocytosis capacity, despite being challenging, holds great promise for boosting therapeutic efficacy in atherosclerosis treatment.

Accumulating evidence has demonstrated that targeted therapy *via* a nanoparticle-mediated drug delivery shows great promise in reducing atherosclerotic burden and plaque inflammation in mice^4^. Previous studies have demonstrated that two-dimensional phosphorene, such as black phosphorus nanosheets, can serve as antioxidants and scavenge a broad range of reactive oxygen species (ROS) to reduce oxidative stress for the treatment of acute kidney injury and atherosclerosis^16^^,^^17^. Here, we discovered that the phosphorene (PP) can function beyond anti-oxidation by enhancing the capacity of macrophage efferocytosis, *via* an enhancement of pro-efferocytosis receptor Mer proto-oncogene tyrosine kinase (MerTK) and a reduction of Ca^2+^/calmodulin-dependent protein kinase *γ* (CaMKII*γ*), and improving M2-like macrophage polarization, showcasing new pleiotropic effects of phosphorene in reducing atherosclerotic burden in mice. Moreover, the surface modification of PP with collagen IV-targeted peptide substantially improve their accumulation in atherosclerotic plaques and lesional macrophages following intravenous administration. Remarkably, the plaque-targeted PP are capable of loading and delivering Resolvin D1 (RD), a potent anti-inflammatory lipid mediator^18^, ^19^, ^20^, ^21^, ^22^, ^23^, to synergistically restore efferocytic capacity of lesional macrophages and enhance the anti-atherosclerosis effect of this nano-formulation (Fig. 1). This study not only presents a nano-immunotherapeutic approach that modulates the efferocytic function of lesional macrophages to alleviate atherosclerotic burden, but also offers a novel strategy for treating diseases related to impaired macrophage efferocytosis.

**Figure 1:**
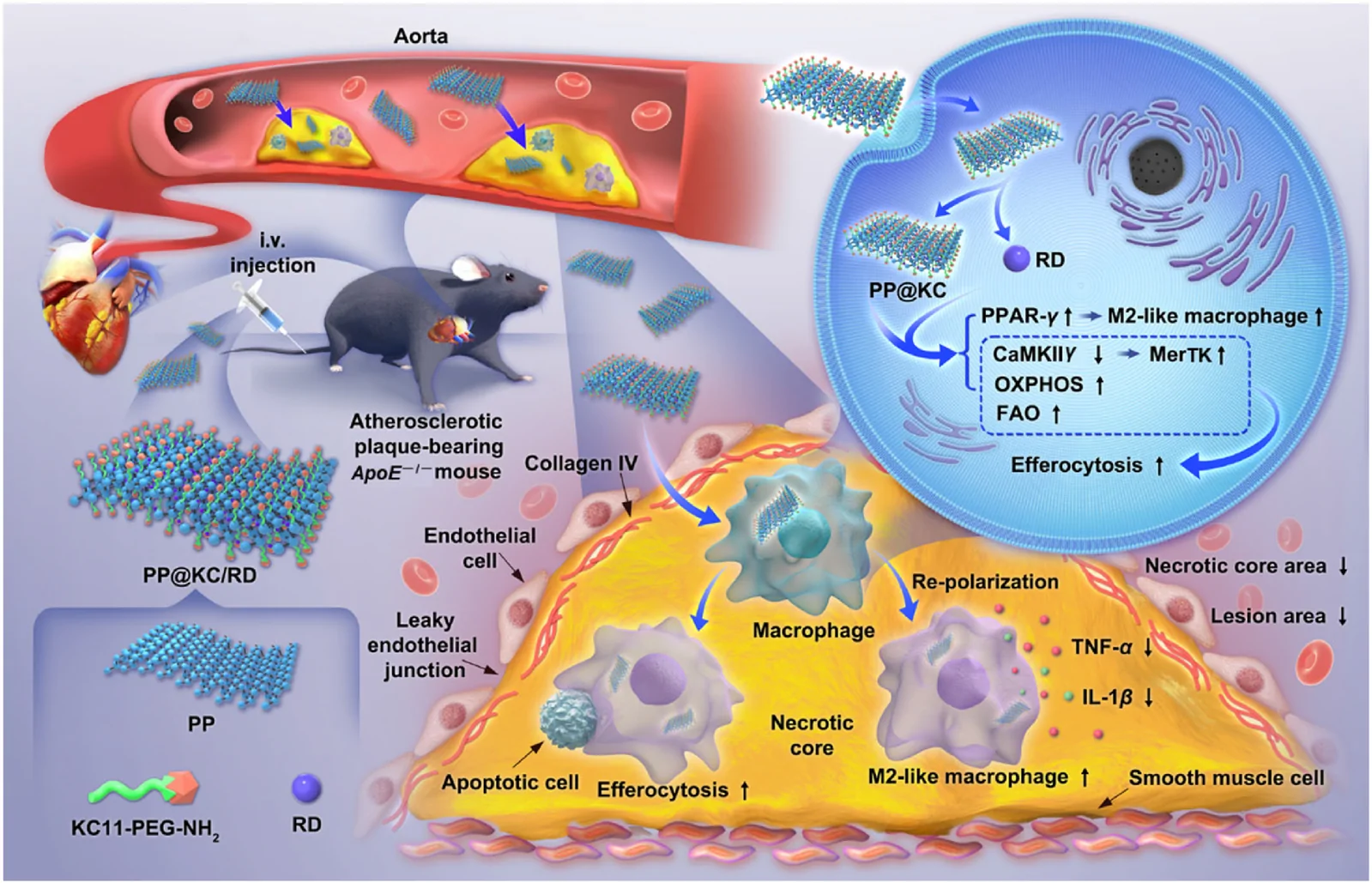
Schematic illustration of the synthetic strategy and anti-atherosclerotic mechanism of PP-based nanomedicine. This study constructed an atherosclerotic plaque-targeting nanoplatform, PP@KC, as a carrier for delivering RD to treat atherosclerosis, and discovered new pleiotropic effects of PP in reducing atherosclerotic burden. The characterization of PP@KC and the loading and release performance of RD were first investigated. The cellular uptake, biocompatibility, enhancement of M2-like phenotype, and restoration of efferocytic capacity of PP@KC/RD were studied in macrophages *in vitro*. The biodistribution and plaque-targeting performance of PP@KC/RD were evaluated in plaque-bearing *ApoE*^−/−^ mice using near-infrared fluorescence imaging, histological, and flow cytometric techniques. Importantly, we elucidated the anti-atherosclerotic efficacy, therapeutic mechanism, and safety profile of PP@KC/RD in atherosclerotic *ApoE*^−/−^ mice. This study first discovered that PP themselves can enhance macrophage efferocytosis *via* an increase in the pro-efferocytosis receptor MerTK, a reduction of CaMKII*γ*, activation of OXPHOS and FAO, and improved M2-like macrophage polarization *via* up-regulation of the PPAR*γ* level, showcasing new pleiotropic effects of PP in reducing atherosclerotic burden in mice. Abbreviations: PP, phosphorene nanosheets; RD, Resolvin D1; CaMKII*γ*, Ca^2+^/calmodulin-dependent protein kinase *γ*; MerTK, Mer proto-oncogene tyrosine kinase; OXPHOS, oxidative phosphorylation; PPAR*γ*, peroxisome proliferator-activated receptor-*γ*; FAO, fatty acid oxidation.

## 2 Materials and methods

### 2.1 Materials and reagents

KC11 peptide (KLWVLPKGGGC) was purchased from Gill Biochemical (Shanghai, China). Mal-PEG-NH_2_, FITC-PEG-NH_2_, and Cy7-NH_2_ were obtained from Ponsure (Shanghai, China). PP crystals were purchased from Smart-Elements (Vienna, Austria) and stored in a dark argon-filled glovebox. ox-LDL and DiI-ox-LDL were obtained from Yiyuan Biotechnologies (Guangzhou, China). Resolvin D1 was purchased from Cayman Chemical (Ann Arbor, Michigan, USA). All mouse ELISA kits were purchased from Solarbio (Beijing, China).

### 2.2 *In vivo* atherosclerosis animal model

Six-week-old male *ApoE*^−/−^ mice with a C57BL/6J background were purchased from fukang Hua biotechnology Co., Ltd. (Beijing, China). The mouse experiments were conducted with review and approval from the West China Hospital at Sichuan University under protocol number 20230928004, and Brigham and Womens's Hospital, Harvard Medical School (USA). To establish the atherosclerosis model, *ApoE*^−/−^ mice were fed a high-fat diet (HFD, 1.25% cholesterol, 20% fat, 45% carbohydrate, and 23% protein, Research Diets Inc., D12108C) for 12 weeks.

### 2.3 *In vivo* bio-distribution study

For *in vivo* bio-distribution studies, atherosclerotic plaque-bearing *ApoE*^−/−^ mice or wild-type C57BL/6J mice were intravenously administered with 200 μL of Cy7, PP@Cy7 (50 μg/mL), or PP@KC/Cy7 (50 μg/mL) with an equivalent Cy7 dosage of 40 μg per mouse. After 12 h, the mice were euthanized, and perfusion with PBS was performed to remove any free Cy7. Subsequently, the aortas and main organs were harvested for visualization and quantitative analysis using an *in vivo* imaging system IVIS, Lumina XR III (PerkinElmer, Waltham, MA, USA). Frozen sections of aortic roots with a thickness of 6 μm, obtained from the treated mice, were incubated overnight at 4 °C with primary antibodies, anti-Col IV antibody (Abcam, ab236640, rabbit, 5 μg/mL) or anti-CD68 antibody (Abcam, ab283654, rabbit, 4 μg/mL) followed by incubation with Alexa Fluor® 488-conjugated goat anti-rabbit IgG secondary antibody (Abcam, ab150077, 4 μg/mL) in the dark at room temperature for 2 h. Next, the stained tissue sections were stained with DAPI to label cell nuclei. Finally, the fluorescence intensities of the tissue sections were observed using a confocal laser scanning microscopy (CLSM), and the quantification was performed using ZEN 2.3 software (blue edition).

### 2.4 Examination of the uptake of cellular specificity of Cy5-labeled PP@KC/RD by flow cytometry *in vivo*

Isolation of single-cell suspensions of aorta, spleen and blood: to investigate cellular specificity, the atherosclerotic plaque-bearin *ApoE*^−/−^ mice were intravenously administered with 200 μL of a consistent dosage of Cy5 (20 μg/mouse) labelled various formulations, including saline, PP@RD/Cy5 (50 μg/mL PP@RD/Cy5 containing 100 ng of RD), or PP@KC/RD/Cy5 (50 μg/mL PP@KC/RD/Cy5 containing 100 ng of RD), twice with a 3-day interval. Three days after the second administration, the treated *ApoE*^−/−^ mice were euthanized, and blood was collected *via* cardiac puncture using EDTA-coated tubes. The mice were then perfused through left ventricular puncture with 20 mL of cold PBS containing heparin (1000 U/mL). The spleens were collected and stored in cold PBS.

The entire aorta was carefully harvested under a dissection microscope, ensuring the aorta and adventitia remained intact. The aortas were then gently cleaned to remove the periaortic fat. Afterward, the aortas were cut into small pieces using dissecting scissors, with at least 100 cuts, homogenized, and then digested using a solution containing collagenase I (Sigma–Aldrich, C0130, 450 U/mL), collagenase XI (Sigma–Aldrich, C7657, 125 U/mL), hyaluronidase (H3506, Sigma–Aldrich, H3506, 60 U/mL), and deoxyribonuclease I (Sigma–Aldrich, D4527, 60 U/mL) in Hank's balanced salt solution at 37 °C for 1 h The resulting aortic cells were filtered through a 70-μm strainer and washed twice to obtain single-cell suspensions in 1 mL of Dulbecco PBS.

Spleens were harvested from the treated *ApoE*^−/−^ mice, and then mashed, filtered through 40-μm cell strainers, incubated with ammonium–chloride–potassium lysis buffer for removal of red blood cells on ice. After 10 min, the splenic cells were washed twice with serum-containing PBS, and then resuspended in 5 mL of Dulbecco's PBS. In addition, the collected blood was incubated with quadruple lysis buffer for 20 min on ice and washed twice with serum-containing PBS. The single-cell suspensions of blood were resuspended in 2 mL of Dulbecco's PBS.

### 2.5 Flow cytometry staining and analysis

The obtained single-cell suspensions from different tissues were counted and diluted to a concentration of 1 × 10^6^ cells per 100 μL. The cells were then incubated with 1 μL of dead/alive dye (BioLegend, Cat. No. 423110, Zombie Red™ Fixable Viability Kit, San Diego, CA, USA) in a dark environment for 15 min. Following this, the aortic, splenic and blood cells were washed with FACS buffer (Dulbecco's PBS supplemented with 2% FBS, 0.5% BSA, and 0.05% NaN_3_), treated with 1.5 μL of FC Block (BD Biosciences, Cat. No. 553141, San Jose, CA, USA). Afterward, the cells of aorta, spleen and blood were stained for 45 min at 4 °C using the corresponding antibodies listed in Supporting Information Tables S1–S3, respectively. For intracellular staining of VSMCs and fibroblast, the aortic cells were fixed and permeabilized using buffers (BD Biosciences, Cat. No. 554714) according to the manufacturer's instructions after extracellular staining. Subsequently, the cells were stained with PE-anti-alpha-smooth muscle actin (Novus, Cat. No. NBP2-34522 PE, 1.5 μg/mL, Centennial, CO, USA), and Alexa Fluor 700-anti-Vimentin (R&D Systems, Cat. No. IC2105N, 1.5 μg/mL, Minneapolis, MN, USA). The Cy5 signal was detected in the APC channel. Data of flow cytometry was acquired using a BD LSRII and a BD LSRFortessa flow cytometer (BD Biosciences), and analysis was conducted using FlowJo v10.0.7 software.

### 2.6 *In vivo* anti-atheroclerotic efficacy

The atherosclerotic plaque-bearing *ApoE*^−/−^ mice were randomly divided into four groups (*n* = 6) and intravenously administrated with the following formulations: (i) saline, (ii) RD, (iii) PP@KC, and (iv) PP@KC/RD *via* the tail vein twice a week for a duration of 12 weeks. The specific conditions for each group were as follows: mice were administrated 150 μL of (i) saline, (ii) saline containing 100 ng of RD, (iii) saline containing 10 μg of PP@KC, (iv) saline containing 10 μg of PP@KC preloaded with 100 ng of RvD1.

After the 12-week treatment, the mice were euthanized, and their entire aortas and aortic roots were harvested. Oil Red O (ORO) staining was performed to evaluate the extent of atherosclerotic lesions. Furthermore, the stability of atherosclerotic plaques was assessed using hematoxylin/eosin (H&E) and Masson's trichrome staining assays. The H&E staining assay allowed for quantification of the necrotic core area, which is defined as the clear area in the stained tissues. Masson's trichrome staining assay was utilized to analyze the morphology and area of collagen in the atherosclerotic lesions. Images of the stained tissues were captured using a Pannoramic® MIDI digital slide scanner, and was quantified using Image-Pro Plus 6.0 software.

### 2.7 Examination of macrophage phenotype in situ

For the examination of macrophage phenotype in aortas, acetone-fixed aortic root sections were permeabilized using PBS containing 2% Triton X-100 for 10 min. Subsequently, the sections were blocked with a solution of PBS containing 5% BSA. After 30 min, the tissue sections were incubated with primary antibodies, including rat monoclonal anti-CD68 antibody (Abcam, Cat. No. ab53444, 1 μg/mL, Cambridge, UK), rabbit monoclonal anti-CD86 antibody (Abcam, Cat. No. ab239075, 3 μg/mL), and rabbit monoclonal anti-CD206 antibody (Abcam, Cat. No. ab64693, 1 μg/mL). After the overnight incubation, the aortic root sections were further stained with the corresponding IgG secondary antibodies: Alexa fluor 488-conjugated goat anti-rat IgG (Cell Signaling Technology, Cat. No. 4416, 4 μg/mL, Danvers, MA, USA) and Alexa fluor 594-conjugated goat anti-rabbit IgG (Affinity Biosciences, Cat. No. S0006, 10 μg/mL, Cincinnati, OH, USA) at room temperature for 2 h. Cell nuclei were stained with DAPI. Finally, the immunostained tissue sections were examined using CLSM (Zeiss LSM880, Carl Zeiss, Oberkochen, Germany), and the quantification was performed using a ZEN 2.3 software (blue edition).

### 2.8 Examination of inflammation of the aortic roots by immunofluorescence staining assay

The tissue sections of aortic root harvested from the treated *ApoE*^−/−^ mice were fixed with ice-cold acetone for 5 min, and then permeabilized using PBS containing 2% Triton X-100 for 10 min. After permeabilization, the aortic root sections were blocked with a solution of PBS containing 5% BSA, and incubated with primary antibodies, including rabbit monoclonal anti-TNF-*α* antibody (Affinity Biosciences, Cat. No. AF7014, 5 μg/mL) and anti-IL-1*β* antibod (Affinity Biosciences, Cat. No. BF0684, 5 μg/mL). After incubation at 4 °C overnight, the aortic root sections were further stained with the corresponding IgG secondary antibodies: Alexa fluor 488-conjugated goat anti-rabbit IgG (Abcam, Cat. No. ab150077, 4 μg/mL) or Alexa fluor 594-conjugated goat anti-rabbit IgG (Affinity Biosciences, Cat. No. S0006, 10 μg/mL) at room temperature for 2 h. Finally, the immunostained tissue sections were tained with DAPI examined using CLSM.

### 2.9 Analysis of efferocytic capacity *in vivo*

To examine the phagocytic clearance of apoptotic cells by macrophages *in vivo*, the aortic root tissue were fixed with ice-cold acetone and stained with TUNEL reagents at 37 °C for 1 h. Subsequently, the incubated sections were blocked with PBS containing 5% BSA for 60 min and incubated with anti-Mac-3 antibody (BD Biosciences, Cat. No. 550292, 0.5 μg/mL) at 4 °C overnight. Afterward, the sections were washed twice, and labeled with Alexa Fluor-conjugated secondary antibodies and counterstained with DAPI. Fluorescence microscope images were captured using CLSM, and the phagocytic index was quantified using Image-Pro Plus 6.0 software to determine the ratio of phagocytosed apoptotic cells to free apoptotic cells.

### 2.10 Statistical analysis

All experiments were conducted at least three times to ensure reproducibility. Data are presented as mean ± standard deviation (SD). The D'Agostino Pearson or Shapiro–Wilk test was first performed to assess the normality of the data. For data of normally distributed, differences between two groups were evaluated using Student's *t*-test (two-tailed). When analyzing differences between multiple comparisons, one-way analysis of variance (ANOVA) with Brown-Forsythe and Welch ANOVA post hoc tests were applied. Additionally, for data of non–normally distributed, we used the Mann–Whitney U test (for two groups) or the Kruskal–Wallis test (for multiple groups) to calculate *P* values. The analyses of significance level were performed using GraphPad Prism 8.0 software (GraphPad, San Diego, CA, USA), and *P* < 0.05 was considered statistically significant.

### 2.11 Additional methods

Other detailed materials and experimental procedures are provided in the Supporting Information, including synthesis of PP nanosheets; synthesis of KC11-PEG-NH_2_; preparation of PP@KC and PP@KC/RD; characterization of PP-based NSs; H_2_O_2_ scavenging capability; degradation of PP@KC in PBS with H_2_O_2_; release of RD from PP@KC/RD; shear-responsive drug release from PP@KC/RD; cell culture; cellular uptake; *in vitro* biocompatibility assay; investigation of macrophage phenotype *in vitro*; anti-inflammatory capability of PP@KC/RD *in vitro*; *in vitro* efferocytosis assay of PP@KC/RD; western blotting assay; quantitative real-time PCR assay; *In vivo* pharmacokinetics and biodistribution evaluation; recruitment of macrophages and Ly-6 Chi cells change; quantitation of pro-inflammatory cytokine in the serum.

## 3 Results

### 3.1 Synthesis and characterization of PP@KC/RD nanoformulation

The PP nanosheets were obtained using a liquid-phase exfoliation strategy by sonicating bulk black phosphorous in water with an ultrasound probe^17^. The size and morphology of the obtained PP were characterized using transmission electron microscopy (TEM), high-resolution transmission electron microscopy (HR-TEM), and atomic force microscope (AFM). TEM and AFM images (Fig. 2A and B) show that the exfoliated PP exhibited a lateral size of approximately 150 nm and a thickness of 9 nm. The HR-TEM image (Fig. 2A) exhibited a lattice spacing of 0.39 nm, which was consistent with the crystalline structure of PP^24^. These observations confirm the successful obtain of PP through the liquid-phase exfoliation strategy^17^.

**Figure 2:**
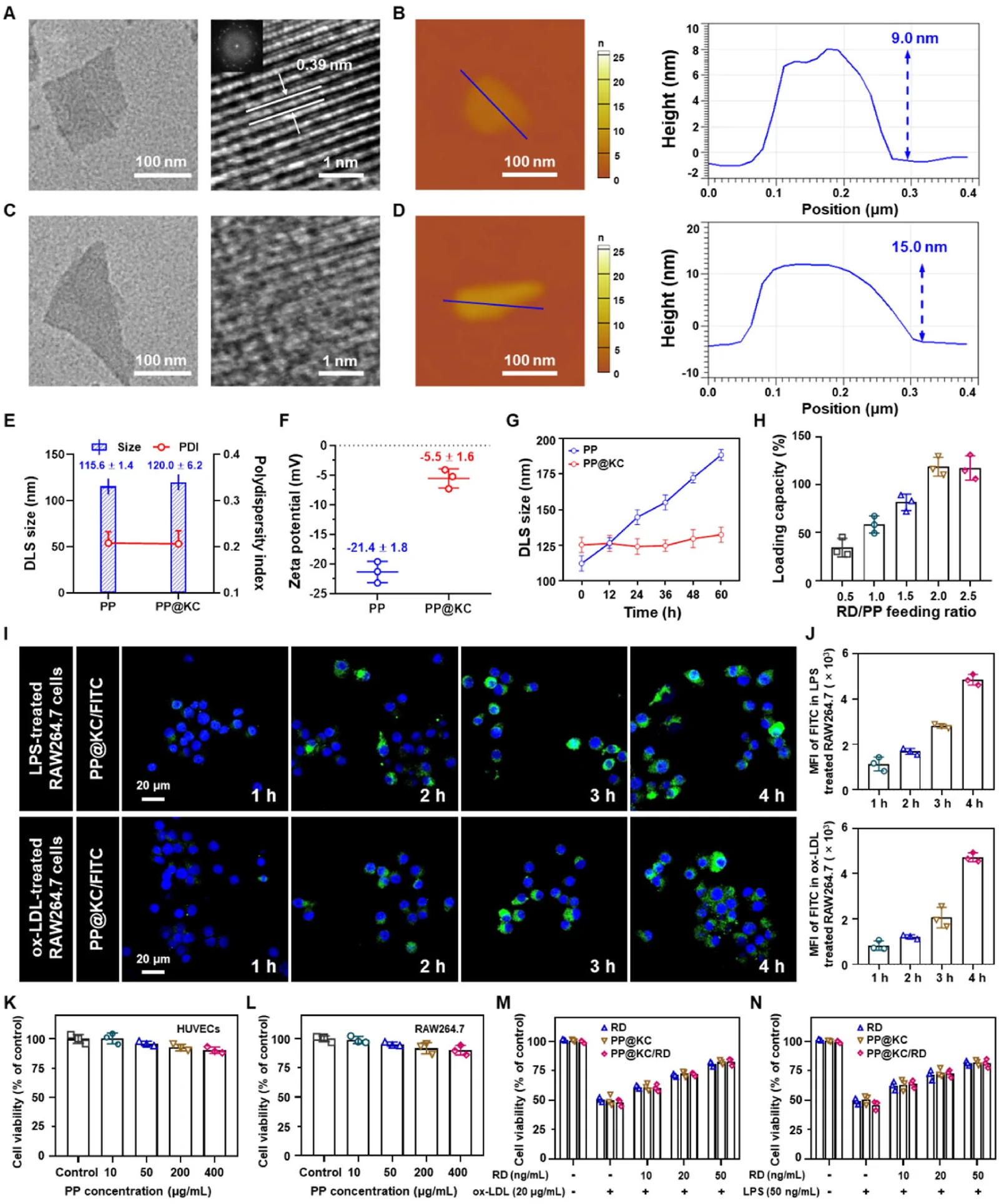
Characterization of PP@KC, RD loading and release performance, and *in vitro* analysis of PP@KC/RD. (A) Morphological characterization of bare PP using TEM (left) and HR-TEM (middle). (B) AFM image and thickness profile of bare PP. (C) TEM (left) and HR-TEM (right) images of PP@KC. (D) AFM image and thickness profile along the blue line in the AFM image. (E) Dynamic light scattering (DLS) sizes and polydispersity index (PDI) of PP and PP@KC (*n* = 3). (F) Change of zeta potential values of PP after KC coating (*n* = 3). (G) Time-course DLS sizes of the nanosheets (*n* = 3). (H) RD loading capacity on PP@KC with different RD/NSs feeding ratios (*n* = 3). (I) Fluorescence microscope images showing the time-dependent cellular uptake of PP@KC/FITC by RAW264.7 cells treated with LPS and ox-LDL, respectively. The cell nuclei (blue) were stained with DAPI. Scale bar = 20 μm. (J) Mean fluorescence intensity (MFI) of FITC-labeled PP@KC in RAW264.7 quantified by flow cytometry analysis (*n* = 3). (K, L) Evaluation of biocompatibility of PP@KC at different PP concentrations (0, 10, 50, 200, and 400 μg/mL) in HUVECs and RAW264.7 cells (*n* = 3). (M, N) Cell viability of RAW264.7 cells co-treated with LPS (50 ng/mL) or ox-LDL (20 μg/mL), along with RD, PP@KC, and PP@KC/RD at the same PP concentrations (50 μg/mL) or different RD concentrations (0, 10, 20, and 50 ng/mL) (*n* = 3). Data are shown as mean ± SD.

To enhance the atherosclerotic plaque-targeting capability of PP and improve their colloidal stability and circulation time *in vivo*, we employed an electrostatic interaction strategy to coat the surface of PP with KC11-PEG-NH_2_ (Fig. 1), which was synthesized through a thioester bond formation reaction between the KC11 peptide and heterobifunctional maleimide-polyethylene glycol-amine (Mal-PEG-NH_2_, Supporting Information Fig. S1). The resulting nanoparticles were designated as PP@KC. The KC11 peptide, consisting of the sequence KLWVLPKGGGC, possesses a strong affinity for collagen IV (Col-IV), which is known to be significantly upregulated in the subendothelial area of inflammatory atherosclerotic plaques^25^, ^26^, ^27^. This specific binding property of KC11 can enhance the accumulation of PP within atherosclerotic plaques following intravenous administration. Compared with naked PP, TEM image of PP@KC revealed that the sheet-like morphology was preserved without significant difference (Fig. 2C). Furthermore, the AFM image showed an increased thickness of PP@KC to 15.0 nm (Fig. 2D), indicating the successful coating of KC11-PEG-NH_2_ on the surface of PP (9.0 nm). It is worth mentioning that the HR-TEM image of PP@KC exhibited blurred lattice spacing (Fig. 2C), providing further evidence that the crystallized PP surface was coated by the KC11-PEG-NH_2_.

Dynamic light scattering (DLS) characterization revealed that the hydrodynamic size of PP@KC is 120.1 ± 6.2 nm, slightly larger than that of PP (115.6 ± 1.4 nm) (Fig. 2E). The zeta potential value of PP@KC (−5.5 ± 1.6 mV) was less negative than that of PP (−21.4 ± 1.8 mV) (Fig. 2F), indicating the successful modification of KC11-PEG-NH_2_ polymer on the PP surface. Furthermore, the size of PP@KC showed no significant change over 60 h in physiological environments, specifically PBS (pH 7.4) supplemented with 10% fetal bovine serum (FBS) and incubated at 37 °C, compared to naked PP (Fig. 2G), confirming that the KC11-PEG-NH_2_ coating successfully improved the colloidal stability of naked PP.

To enhance the anti-atherosclerosis effect of PP@KC, we loaded RD, a potent pro-resolving lipid mediator that is typically deficient in vulnerable atherosclerotic plaques^18^, onto the surface of PP@KC. The resulting formulation, named PP@KC/RD, exhibited efficient loading of RD with a loading capacity of 118.7 (%, *w*/*w*) at an RD/PP@KC feeding ratio of 2 (Fig. 2H). TEM image revealed that the sheet-like structure and average size of PP@KC remained largely unchanged after RD loading (Supporting Information Fig. S2A). The slight increase in PP@KC/RD thickness further confirmed the successful surface loading of RD (Fig. S2B). Furthermore, the Raman spectrum of PP@KC/RD exhibited a slight red shift compared to PP, while retaining the prominent peaks at approximately 363 cm^−1^ (*A*_1g_), 439 cm^−1^ (*B*_2g_), and 468 cm^−1^ (*A*_2g_) (Fig. S2C). This observation can be attributed to the efficient binding between PP, KC11-PEG-NH_2_ and RD. Additionally, the diffraction peaks of X-ray diffraction pattern of PP@KC/RD matched those of the standard orthorhombic PP (JCPDS #73-1358), indicating the retention of the crystalline structure after KC and RD loading (Fig. S2D)^28^. Furthermore, the hydrodynamic size of PP@KC/RD exhibited no significant changes in PBS and cell culture medium (DMEM with 10% FBS) even after 72 h, demonstrating superior colloidal stability of the drug-loaded nanocarriers in physiological environments (Fig. S2E). Moreover, the reactive oxygen species (ROS)-responsive release of RD from PP@KC/RD has been demonstrated (Fig. S2F–S2H)^17^. In order to examine RD release behavior under simulated shear flow to better predict *in vivo* drug availability and release dynamics. A parallel-plate flow chamber with PP@KC/RD on collagen-IV glass was used to apply oscillatory shear stress (OSS, 0.5 dyn cm^−2^), low laminar shear stress (LSS, 12 dyn cm^−2^), and high shear stress (HSS, 30 dyn cm^−2^) for 24 h, showing cumulative RD release increased from 87% (LSS) to 95% (HSS), indicating local blood flow enhances drug delivery in atherosclerotic lesions (Supporting Information Fig. S3). The remarkable loading capacity of RD, excellent colloidal stability, and ROS-responsiveness of PP@KC not only minimizes RD premature release in the normal physiological microenvironment (PBS pH 7.4 supplemented with 10% FBS and incubated at 37 °C), but also enhances the drug concentration in atherosclerotic plaques, highlighting the potential of PP@KC as effective nanocarriers for drug delivery and achieving synergistic treatment of atherosclerosis.

### 3.2 *In vitro* studies of cellular uptake and biocompatibility of PP@KC/RD

To assess the cellular uptake efficiency, we used FITC-labeled PP (PP@FITC) and FITC-labeled PP@KC (PP@KC/FITC) and monitored their uptake using a confocal laser scanning microscope (CLSM). The results revealed a time-dependent increase in green fluorescence around the nuclei (blue) of LPS-treated or oxidized low-density lipoprotein (ox-LDL)-treated RAW264.7 macrophages, indicating enhanced uptake of PP@KC/FITC over time (Fig. 2I and J, and Supporting Information Figs. S4–S6).

We also assessed the biocompatibility of PP@KC and observed no cytotoxicity in human umbilical vein endothelial cells (HUVECs, Fig. 2K) and RAW264.7 macrophages (Fig. 2L) even at a high concentration of 400 μg/mL for 24 h. Furthermore, the various formulations, including RD, PP@KC, and PP@KC/RD, showed dose-dependent improvements in LPS or ox-LDL-stimulated macrophages (Fig. 2M and N)^29^. These findings indicate the biocompatibility of the nanoplatforms and support their potential for further anti-atherosclerosis studies.

### 3.3 Macrophage polarization by PP@KC/RD treatment

Next, we investigated whether PP@KC/RD treatment can enhance the polarization of macrophages towards the M2-like subtype that participates in anti-inflammatory response. Using LPS-treated RAW264.7 cells as a model for pro-inflammatory macrophages in atherosclerotic plaques^30^, flow cytometry analysis revealed that PP@KC/RD treatment showed the highest M2-like macrophage polarization with CD206^+^ expression (30.9%) compared to LPS (14.6%), RvD1 (21.0%), and PP@KC (21.8%) treatments (Fig. 3A and C, and Supporting Information Fig. S7). Conversely, the population of M1-like phenotype, characterized by CD86^+^ expression significantly decreased after treatment with RD (28.7%), PP@KC (25.7%), and PP@KC/RD (23.5%). Moreover, the ratio of CD206^+^/CD86^+^ macrophages after PP@KC/RD treatment was approximately 3.8, 1.9, and 1.5 times higher than those of control, RD, and PP@KC treatments, respectively. Additionally, CLSM images (Fig. 3B) and quantitative analysis (Fig. 3D) demonstrated that PP@KC/RD-treated macrophages exhibited the highest CD206 expression compared to RD and PP@KC treatment alone, while exhibiting the lowest CD86 expression compared with other groups. These findings collectively demonstrate that PP@KC/RD treatment effectively promotes the polarization of M2-like macrophages, holding great promise for anti-atherosclerosis treatment.

**Figure 3:**
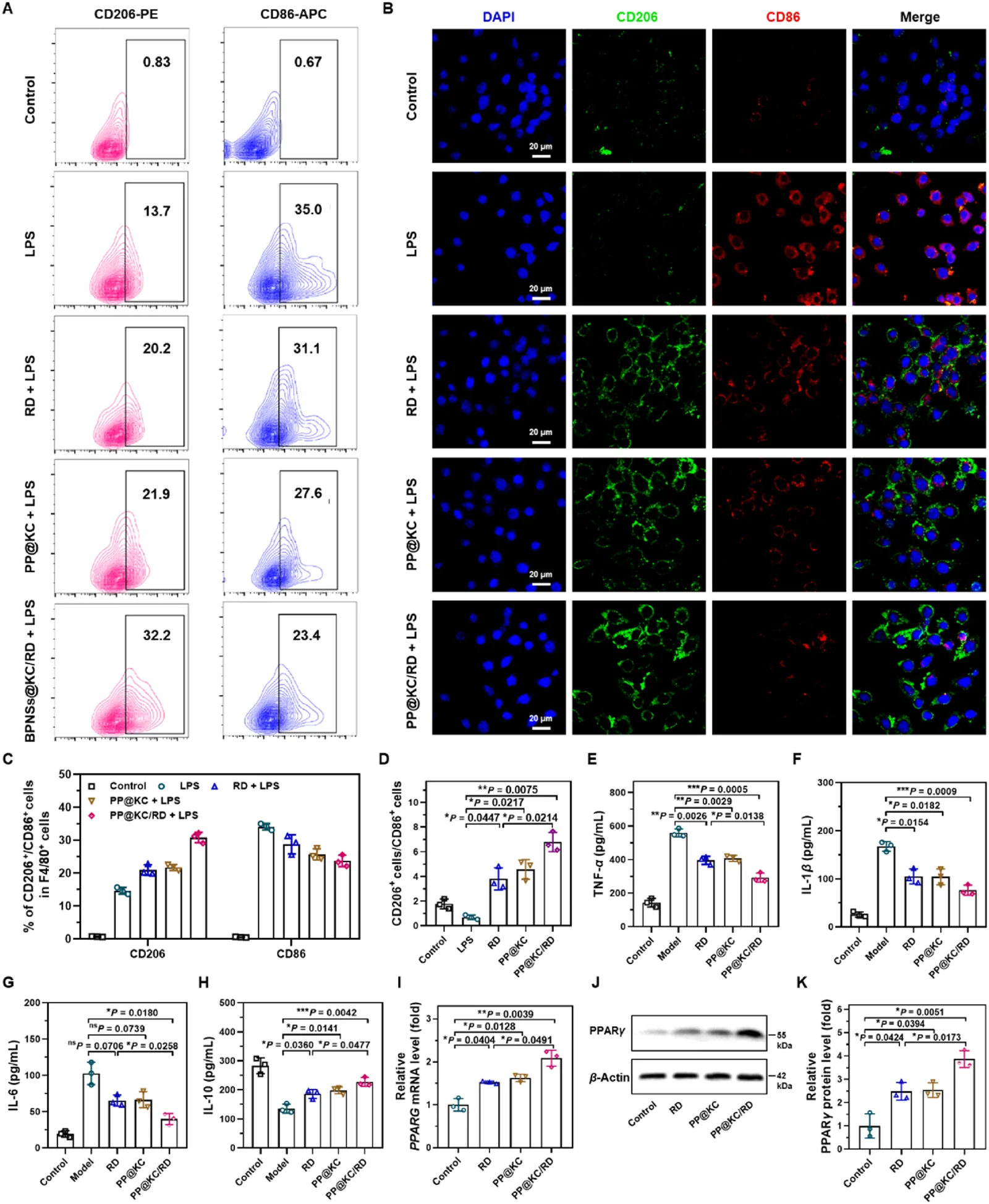
Induction of M2-like macrophage polarization by PP@KC/RD. (A, B) Flow cytometry analysis and fluorescence microscope images showing CD206^+^ or CD86^+^ macrophages in RAW264.7 cells treated with various formulations. Red: CD86 antibody staining for M1-like macrophages; Green: CD206 antibody staining for M2-like macrophages; Blue: DAPI staining for cell nuclei. Scale bar = 20 μm. (C) Quantitative analysis of CD206^+^ or CD86^+^ cell expression in RAW264.7 cells from panel (A) (*n* = 3). (D) Quantification of the CD206^+^/CD86^+^ ratio from panel (B) (*n* = 3). (E–G) Secretion concentration of TNF-*α*, IL-1*β*, and IL-6, and (H) IL-10 by RAW264.7 cells (*n* = 3). The cells were pretreated with medium (model) or various formulations (RD, PP@KC, and PP@KC/RD) for 2 h, followed by co-incubation with LPS (500 ng/mL) for 12 h. The concentrations of cytokines were measured using an ELISA assay. Control: cells without any treatment (I). Relative mRNA expression of PPAR-*γ* genes in LPS-stimulated RAW264.7 cells pretreated with various formulations evaluated by RT-qPCR (*n* = 3). (J, K) Western blot analysis and quantification of PPAR-*γ* protein expression in LPS-stimulated RAW264.7 cells pretreated with various formulations (*n* = 3). All data are expressed as mean ± SD. Statistical significance was analyzed using one-way ANOVA with Dunnett's T3 *post hoc* test. ∗*P* < 0.05, ∗∗*P* < 0.01, ∗∗∗*P* < 0.001, and *P* > 0.05 indicates no significance.

To further evaluate the effectiveness of PP@KC/RD in treating atherosclerosis, anti-inflammatory studies using RAW264.7 macrophages were performed. RAW264.7 macrophages were treated by various conditions, including RD, PP@KC, and PP@KC/RD. Compared with the model group treated with LPS alone, the secretion of pro-inflammatory cytokines, including TNF-*α*, IL-6, and IL-1*β*, by RAW264.7 macrophages was moderately reduced in both the RD and PP@KC-treated groups (Fig. 3E–G). Importantly, the PP@KC/RD treatment significantly decreased the secretion of these pro-inflammatory cytokines by RAW264.7 macrophages, compared to RD or PP@KC alone, demonstrating a combination anti-inflammatory effect. Moreover, the PP@KC/RD treatment substantially increased the secretion of anti-inflammatory cytokine IL-10 by RAW264.7 macrophages (Fig. 3H). These results demonstrate that PP@KC/RD effectively reduces the secretion of pro-inflammatory cytokines and increases anti-inflammatory cytokines upon M2-like macrophage polarization.

Subsequently, we investigated the underlying mechanism of improved polarization of M2-like macrophages following PP@KC/RD treatment. The expression of peroxisome proliferator-activated receptor-*γ* (PPAR-*γ*), known to specifically activate M2-like macrophage polarization was studied^31^^,^^32^. The reverse transcription-quantitative polymerase chain reaction (RT-qPCR) assay (Fig. 3I) revealed that the mRNA level of PPAR-*γ* in PP@KC/RD-treated macrophages was markedly higher than in the other cohorts. Consistent with the gene expression results, Western blot analysis demonstrated a substantial up-regulation of PPAR-*γ* protein expression in PP@KC/RD-treated macrophages (Fig. 3J and Supporting Information Table S4). The protein expression of PPAR-*γ* was 3.8, 1.5, and 1.5 times higher than that of the control, RD, and PP@KC groups, respectively (Fig. 3K). These combined results suggest that both RD and PP@KC treatments moderately promoted M2-like macrophage polarization through the activation of the PPAR-*γ* pathway. Importantly, treatment with PP@KC/RD significantly upregulated the levels of PPAR-*γ* mRNA and protein expression in macrophages, leading to a substantial promotion of M2-like macrophage polarization through the combination activation of RD and PP@KC. Collectively, these findings demonstrate that PP@KC/RD nanotherapeutics have the capability to effectively polarize pro-inflammatory macrophages towards an anti-inflammatory phenotype through the activation of the PPAR-*γ* pathway.

### 3.4 Restoration of macrophage efferocytic capacity by PP@KC/RD

Next, we investigated the potential of PP@KC/RD to restore phagocytic functions of macrophages, which could lead to improved clearance of apoptotic cells in plaques and contribute to the regression of atherosclerotic plaques^7^, ^8^, ^9^. An *in vitro* phagocytosis assay showed that, compared to the treatments with RD or PP@KC alone, PP@KC/RD treatment significantly enhanced the phagocytic capacity of RAW264.7 cells (green, Fig. 4A and Supporting Information Fig. S8) against apoptotic macrophages (red, Fig. 4A and Fig. S8) that had been exposed to the pro-atherosclerostic tumor necrosis factor-*α* (TNF-*α*). This finding demonstrates that the treatment effectively improves the efferocytic capability of RAW264.7 macrophages.

**Figure 4:**
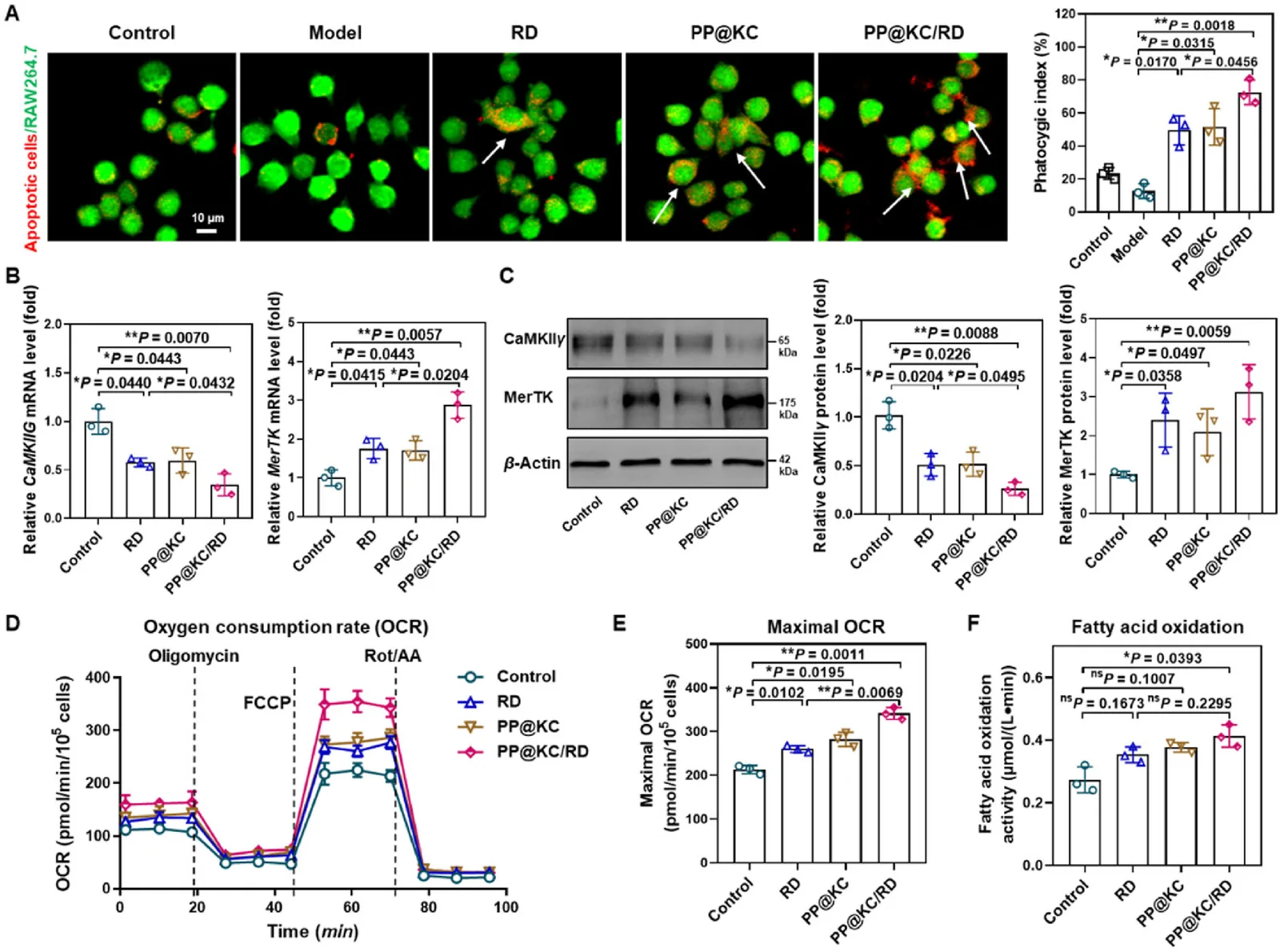
Analysis of RAW264.7 cell efferocytosis after PP@KC/RD treatment. (A) Phagocytosis efficiency of macrophages (CellTracker Green^+^) against apoptotic cells (CellTracker Red^+^). RAW264.7 cells were preincubated with various formulations for 2 h and labeled with CellTracker Green for 30 min. They were then co-incubated with CellTracker Red–labeled apoptotic cells at a macrophage-to-apoptotic cell ratio of 1:3 for 2 h. The phagocytosis assay was captured by confocal laser scanning microscopy (Left). Quantification results of macrophage efferocytosis (right). Yellow overlays (arrows) indicate macrophages with internalized apoptotic cells (uptake) (*n* = 3). Scale bar = 10 μm. (B) Relative mRNA expression of CaMKII*γ* and MerTK genes evaluated by RT-qPCR in LPS-stimulated RAW264.7 cells pretreated with various formulations. Cells treated with LPS only serves as the control (*n* = 3). (C) Protein expression of CaMKII*γ* and MerTK examined and quantified by western blotting in LPS-stimulated RAW264.7 cells pretreated with various formulations (*n* = 3). Cells treated with LPS only serve as the control. (D) Representative trace of normalized oxygen consumption rate (OCR) in RAW264.7 cells treated with various formulations. Oxidative phosphorylation (OXPHOS) was quantified by monitoring OCR with an XF-24 Extracellular Flux Analyzer following sequential treatments with oligomycin (oligo), FCCP, and rotenone/antimycin A (Rot/AA). (E) Quantitative analysis of maximal OCR in Figure (D) (*n* = 3). (F) Fatty acid oxidation assay in apoptotic cell-stimulated macrophages treated with various formulations (*n* = 3). All data are expressed as mean ± SD Statistical significance was analyzed using one-way ANOVA with Dunnett T3 *post hoc* test. ∗*P* < 0.05, ∗∗*P* < 0.01, and *P* > 0.05 indicate no significance.

To investigate the underlying mechanism of enhanced macrophage efferocytic capacity by PP@KC/RD, we further examined the expression of Ca^2+^/calmodulin-dependent protein kinase *γ* (CaMKII*γ*) gene and protein, which can suppress the expression of the pro-efferocytosis receptor Mer proto-oncogene tyrosine kinase (MerTK), leading to impaired efferocytosis and exacerbation of plaque necrosis in atherosclerotic lesions^33^^,^^34^. RT-qPCR assay results showed that PP@KC/RD treatment significantly reduced the mRNA expression level of CaMKII*γ*, NF-*κ*B, and MAPK and markedly increased the mRNA expression level of MerTK in macrophages (Fig. 4B, Supporting Information Fig. S9, and Table S4), demonstrating that PP@KC/RD effectively improved efferocytosis. Consistently, protein analysis revealed similar trends in the levels of CaMKII*γ* and MerTK after PP@KC/RD treatment (Fig. 4C), further supporting the pro-efferocytic PP@KC/RD for apoptotic cell clearance.

Furthermore, additional mechanistic studies revealed that PP@KC/RD treatment promoted oxidative phosphorylation (OXPHOS) and fatty acid oxidation (FAO) in macrophages (Fig. 4D–F). These metabolic changes are known to activate macrophage efferocytosis and reduce the accumulation of necroptotic cells^35^. Collectively, these findings indicate that PP@KC/RD treatment significantly enhances the phagocytic capability of macrophages by downregulating the CaMKII*γ* pathway and activating OXPHOS and FAO.

### 3.5 *In vivo* targeting capability of PP@KC

We next evaluated the *in vivo* plaque-targeting capability of PP@KC in atherosclerotic *ApoE*^−/−^ mice fed a high-fat diet (HFD) for 12 weeks^17^^,^^36^. Intravenous administration of free Cy7, Cy7-conjugated PP (PP@Cy7), or Cy7-conjugated PP@KC (PP@KC/Cy7) was performed, and aortas were harvested 12 h after injection. The fluorescent signals of Cy7 in the aorta were approximately 4.3 times higher for targeted PP@KC/Cy7 than for non-targeted PP@Cy7 (Fig. 5A and B). In comparison, the fluorescent signals in wild-type mice treated with PP@KC/Cy7 were much lower than those in the atherosclerotic mice. These results indicate the effective accumulation of targeted PP@KC/Cy7 in the lesional areas of atherosclerotic mice. In addition, We used Cy5 fluorescence labeling to evaluate the *in vivo* behavior of RD following intravenous administration, with plasma fluorescence measured at various time points. The resulting plasma concentration–time profile exhibited two-compartment pharmacokinetics, with a half-life of 6.8 h for the nanomedicine, which was significantly longer than that of free Cy5/RD (1.5 h), indicating that the nanocarrier extends the circulation time of RD (Supporting Information Fig. S10A). *Ex vivo* imaging at 24 h revealed accumulation in the liver and spleen, with minimal signals in the heart, lungs, and kidneys (Fig. S10B).

**Figure 5:**
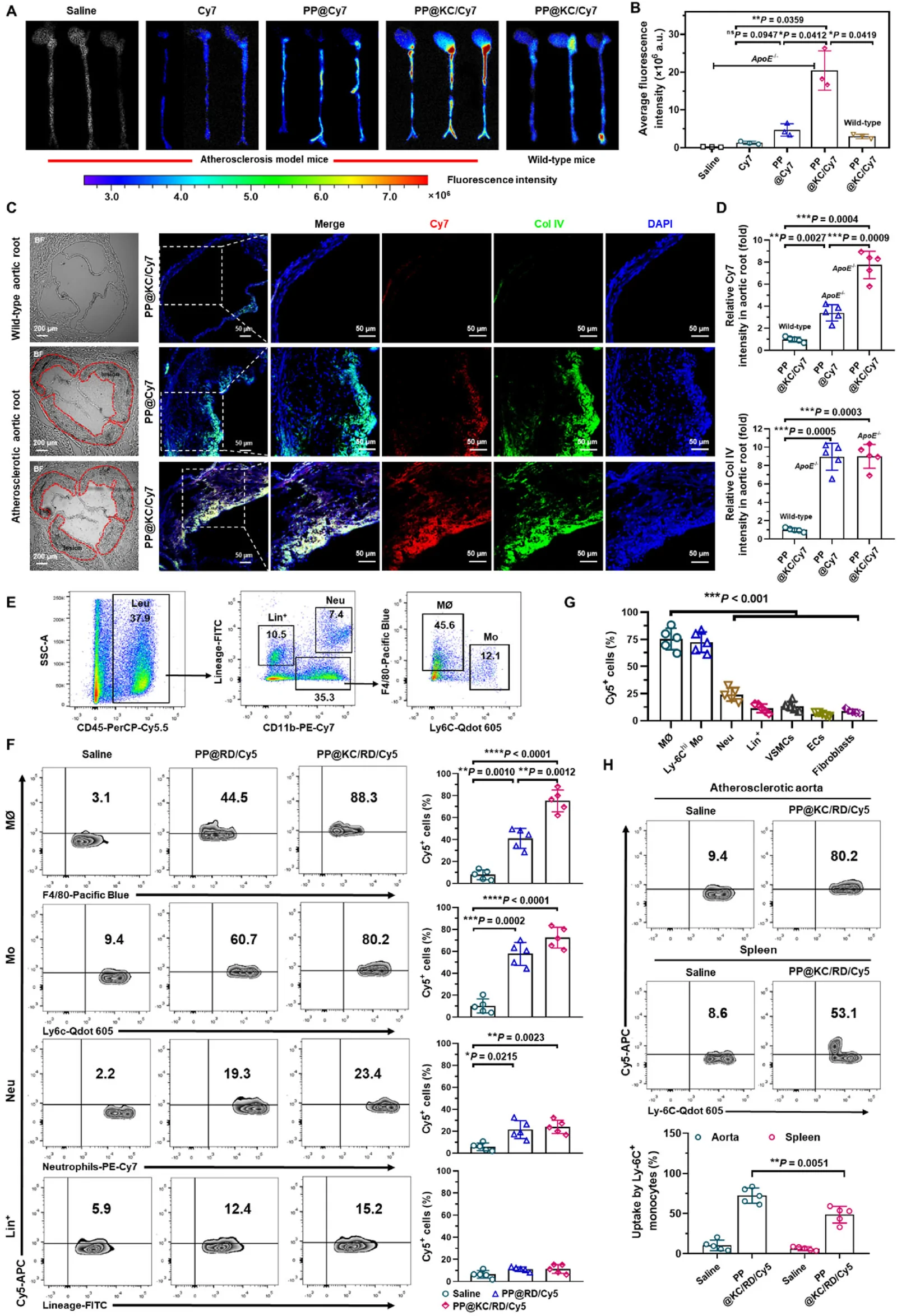
*In vivo* plaque-targeting performance of PP@KC/RD. (A, B) Representation of IVIS images and quantitative analysis of Cy7 fluorescence signals in the aorta of plaque-bearing *ApoE*^−/−^ mice or C57BL/6J wild-type mice. The mice were sacrificed 12 h post-injection of saline, free Cy7, PP@Cy7, or PP@KC/Cy7, 40 μg of Cy7 per mouse (*n* = 3). (C) CLSM images of aortic root sections from plaque-bearing *ApoE*^−/−^ mice treated with PP@KC/Cy7 or PP@Cy7, and C57BL/6J wild-type mice treated with PP@KC/Cy7. Blue: cell nuclei stained with DAPI; Green: Collagen IV (Col IV); Red: Cy7-labeled PP or PP@KC. Scale bar = 50 or 200 μm. (D) Quantification of the fluorescence intensity ratio of Cy7 and Col IV in the aortic root sections shown in (C) (*n* = 5). (E) Gating strategy used in the flow cytometry evaluation of vascular cell specificity using targeted PP@KC/RD/Cy5, and non-targeted PP@RD/Cy5 in plaque-bearing *ApoE*^*−/−*^ mice. A detailed gating strategy is provided in the Supporting Information M_*Ø*_ = macrophages; Mo = Ly-6C^hi^ monocytes; Neu = neutrophils; Lin^+^ = lineage-positive CD11b-negative cells; Leu = leukocytes. (F) Representative flow cytometry contour plot and quantitative analysis of the Cy5^+^ cells in different cell types from *ApoE*^−/−^ mice aortas following administration of saline, PP@RD/Cy5, and PP@KC/RD/Cy5 (*n* = 5). (G) PP@KC/RD/Cy5 specifically accumulate within macrophages and Ly-6C^hi^ monocytes in the atherosclerotic aorta, while detection of PP@KC/RD/Cy5 in other vascular cells is low (*n* = 5). (H) Comparison of the accumulation of Ly-6C^hi^ monocytes in the aorta and the spleen (*n* = 5). All graphs present data as mean ± SD. Data were analyzed using one-way ANOVA with a Dunnett T3 *post hoc* test (in panels B and G), a Games-Howell *post hoc* test (in panels D and F), or by unpaired two-tailed Student's *t*-test (in panel H) ∗*P* < 0.05, ∗∗*P* < 0.01, and ∗∗∗*P* < 0.001, while *P* > 0.05 denotes no significance.

To further investigate the enhanced accumulation attributed to the specific binding between the KC11 targeting peptide and overexpressed Col IV in the lesional areas, sections of the aortic root harvested from atherosclerotic or healthy aortas were stained with Col IV and DAPI. Immunofluorescence staining assay revealed significantly higher fluorescent signals of Cy7 in the lesional areas of PP@KC/Cy7-treated atherosclerotic mice compared with wild-type mice (Fig. 5C and D). Additionally, most of the Cy7 signals in the lesional plaques overlapped with the Col IV signals, as indicated by the yellow signals in the merged images (Fig. 5C). In contrast, the fluorescent signals of Cy7 in the lesional areas of PP@Cy7-treated atherosclerostic mice were much lower than those of targeted PP@KC/Cy7 (Fig. 5C and D). These findings demonstrate that PP@KC/Cy7 effectively filtrates the arterial walls and reaches the lesional plaques, with the KC11 targeting peptide facilitating the enhanced accumulation of the drug-loaded nanotherapeutics primarily through effective targeting between KC11 peptide and overexpressed Col IV in the lesional area.

To further study the nanoparticle uptake by various vascular cells in atherosclerotic *ApoE*^−/−^ mice, we conducted a comprehensive analysis using ten-color flow cytometry after intravenous administration of Cy5-labeled PP@KC/RD (PP@KC/RD/Cy5) (Supporting Information Fig. S11 and Table S2). The results revealed that the majority of PP@KC/RD/Cy5 were effectively taken up by lesional macrophages (∼76%) and Ly-6C^hi^ monocytes (∼72%), much higher than the neutrophils (∼24%), VSMCs (∼13%) and lineage-positive cells (∼11%) after two biweekly PP@KC/RD/Cy5 administration (Fig. 5E–G). Minimal amounts of PP@KC/RD/Cy5 were detected in endothelial cells (∼6%) and fibroblasts (∼8%) (Fig. 5F and G, and Fig. S11). Moreover, the accumulation of PP@KC/RD/Cy5 within lesional macrophages was significantly higher than that observed with non-targeted PP/RD/Cy5 treatment (76% *versus* 41%, Fig. 5F and Fig. S11). Consistently, confocal fluorescence imaging displayed a notable co-localization of Cy7 fluorescent signals from targeted PP@KC/Cy7 (Cy7, red) with lesional macrophages (CD68, green) in the aortic root section, with approximately 2.6-fold higher fluorescence intensity compared to non-targeted PP/Cy7 treatment (Supporting Information Fig. S12).

Moreover, an additional observation revealed that a greater percentage of Ly-6C^hi^ monocytes had taken up PP@KC/RD/Cy5 in the atherosclerotic aorta compared to the spleen (72% *versus* 48%, Fig. 5H, Supporting Information Figs. S13 and 14, and Table S3). This finding indicates that, apart from the enhanced plaque accumulation resulting from the specific binding between the KC targeting peptide of PP@KC/RD and overexpressed Col IV receptors in atherosclerotic lesional plaques and macrophages within the arterial wall, PP@KC/RD may be efficiently delivered to the diseased artery through circulating inflammatory monocytes (Fig. S14 and Table S4)^37^. Subsequently, these PP@KC/RD-loaded monocytes migrate to the inflammatory atherosclerotic plaques. These results demonstrate that intravenous administration of targeted PP@KC/RD not only effectively infiltrates atherosclerotic plaques but also enhances their accumulation within lesional macrophages. Moreover, plaque accumulation of PP@KC/RD is primarily driven by KC11-mediated affinity targeting to the lesion microenvironment, rather than by altered macrophage recruitment dynamics or Ly-6C^hi^ monocyte–dependent hitchhiking. This finding further supports the specificity of the targeting strategy employed in this system (Supporting Information Fig. S15).

### 3.6 *In vivo* therapeutic efficacy of PP@KC/RD in atherosclerotic mice

After confirming the enhanced plaque-accumulating capability of PP@KC, we next examined the anti-atherosclerotic efficacy of PP@KC/RD *in vivo* (Fig. 6A). Atherosclerotic plaque-bearing mice (*ApoE*^−/−^ mice fed a HFD for 12 weeks) were intravenously injected with saline, RD, PP@KC, or PP@KC/RD twice a week for an additional 12 weeks while being maintained on the HFD. To quantify the areas of atherosclerotic plaques, the entire aortas and aortic roots were harvested and stained by Oil Red O (ORO) one week after the last administration^38^; the red regions in the tissues represent the atherosclerotic lesion area (Fig. 6B and C). Analysis of the stained tissues revealed that RD- or PP@KC-treated atherosclerotic mice showed moderate therapeutic efficacy, with the ratios of lesion area to the entire aorta area decreasing to 21.1% and 17.8%, respectively, compared to 32.4% in the saline group (Fig. 6B and F). Remarkably, the PP@KC/RD cohorts exhibited a significant decrease in lesion areas (9.4%, Fig. 6B and F). These findings not only demonstrate the effective delivery of RD to the lesion area *in vivo* using PP@KC/RD, but also highlight the enhanced therapeutic efficacy of the PP@KC nanocarriers and RD, resulting in superior therapeutic efficacy for atherosclerosis treatment. Additionally, analysis of the lesion areas in the aortic roots of the treated mice (Fig. 6C–G, and Supporting Information Fig. S16A) aligns with the findings in the entire aorta, showing moderate therapeutic efficacy for RD and PP@KC treatment and the most effective therapeutic efficacy for the PP@KC/RD cohort, with the lesion area reduced to approximately 25% compared to the saline control group.

**Figure 6:**
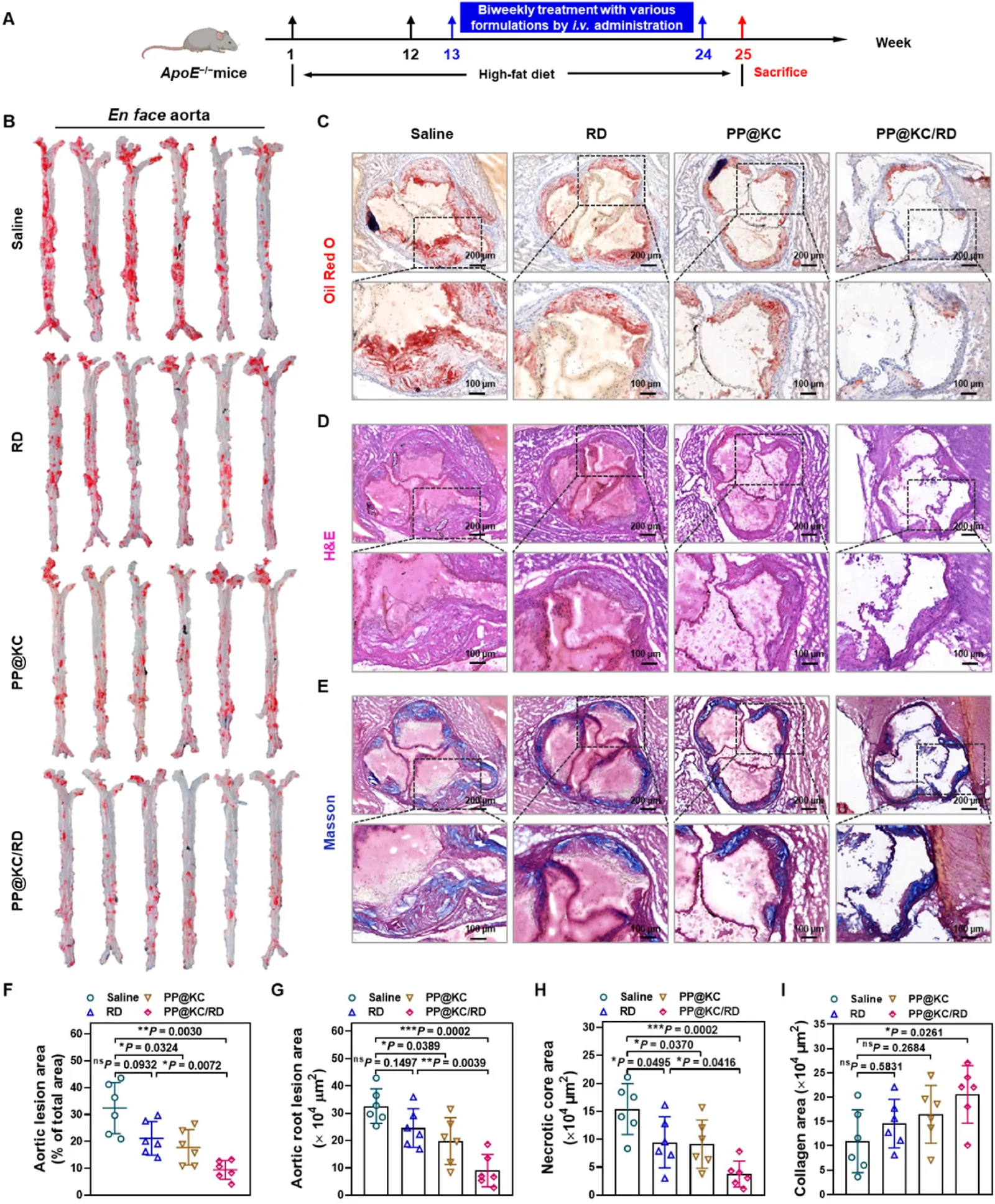
*In vivo* therapeutic efficacy of PP@KC/RD in *ApoE*^−/−^ mice. (A) Schematic illustration depicting the establishment of atherosclerotic mice and the treatment protocol. Plaque-bearing *ApoE*^−/−^ mice were intravenously treated with 150 μL of saline, RD (100 ng of RD/mouse), PP@KC (10 μg of PP@KC/mouse), and PP@KC/RD (10 μg of PP@KC preloaded with 100 ng of RD/mouse). (B) *En face* entire aortas stained with Oil Red O (ORO) to analyze atherosclerotic lesion area. (C–E) Representative images of (C) ORO (D) H&E, or (E) Masson's trichrome stained aortic root sections from mice after various treatments. Scale bar = 100 or 200 μm. (F) Quantitative analysis of the ratio of ORO-positive lesion area to the entire aorta in (B). (G–I) Quantitative analysis of (G) total ORO-positive lesion area (H) necrotic area, and (I) collagen area in the atherosclerotic plaques of the aortic root sections in (C) (*n* = 6). For panels (F) and (G), data were analyzed using one-way ANOVA with a Dunnett T3 *post hoc* test. For panels (H) and (I), data were analyzed using one-way ANOVA with a Tukey *post hoc* test. Data are presented as mean ± SD ∗*P* < 0.05, ∗∗*P* < 0.01, ∗∗∗*P* < 0.001 and ns denotes no significance.

To assess the potential enhancement of stability in the atherosclerotic plaques following various treatments, we utilized hematoxylin/eosin (H&E) and Masson's trichrome staining assays to examine the necrotic core area and collagen cap thickness of the atherosclerotic lesions^39^. The PP@KC/RD cohort exhibited a reduction in the necrotic core area (Fig. 6D and H), and the ratio of necrotic core area to lesion size (Fig. S16B), indicating improved plaque stability. Additionally, the collagen cap area and the ratio of collagen cap area to lesion size were enhanced (Fig. 6E and I, and Fig. S16C), further supporting increased plaque stability. These findings demonstrate that treatment with PP@KC/RD effectively reduces plaque areas and significantly enhances plaque stability in HFD-fed *ApoE*^−/−^ mice, highlighting the promise of the PP@KC/RD nanotherapeutics for atherosclerosis treatment.

### 3.7 Anti-atherosclerotic mechanism of PP@KC/RD *in vivo*

To unravel the potential mechanisms underlying the remarkable therapeutic efficacy of PP@KC/RD, we investigated two key aspects: (i) the polarization of lesional macrophages and its impact on anti-inflammation, and (ii) the effect on efferocytic capacity on lesional macrophages. Given that M2-like macrophages possess the capability to resolve inflammation^12^^,^^13^, we designed a specific study to examine the phenotypes of lesional macrophages in the plaques *in vivo* following various treatments.

Immunostaining assay revealed that PP@KC/RD treatment resulted in the highest levels of CD68^+^CD206^+^ signals (indicative of M2-like macrophages) in the aortic root sections from plaque-bearing *ApoE*^−/−^ mice (Fig. 7A and B). Conversely, the fluorescence intensity of CD68^+^CD86^+^ (indicative of M1-like macrophages) in the lesion areas was markedly decreased following treatment with PP@KC/RD (Fig. 7A and C). Collectively, these findings from both *in vitro* and *in vivo* studies demonstrate that PP@KC/RD effectively polarizes macrophages toward an M2 phenotype, which can execute anti-inflammatory activities. Furthermore, immunofluorescence images demonstrated that treatment with PP@KC/RD exhibited the attenuated the levels of pro-inflammatory cytokines, including TNF-*α* (green, Fig. 7D, Supporting Information Fig. S17A) and IL-1*β* (red, Fig. 7D, and Fig. S17B), compared to other treatment groups. These results indicate that PP@KC/RD effectively resolves inflammation in the lesion area. Additionally, we measured the concentrations of TNF-*α* and IL-1*β* in the serum to assess systematic inflammation in the treated *ApoE*^−/−^ mice. Consistent with the immunofluorescence findings in the aortic roots, PP@KC/RD treatment demonstrated the lowest serum levels of TNF-*α* (Fig. 7E) and IL-1*β* (Fig. 7F). These results align with the *in vitro* studies, illustrating that PP@KC/RD exhibits desirable inflammation resolution capabilities, thereby achieving remarkable anti-atherosclerosis efficacy.

**Figure 7:**
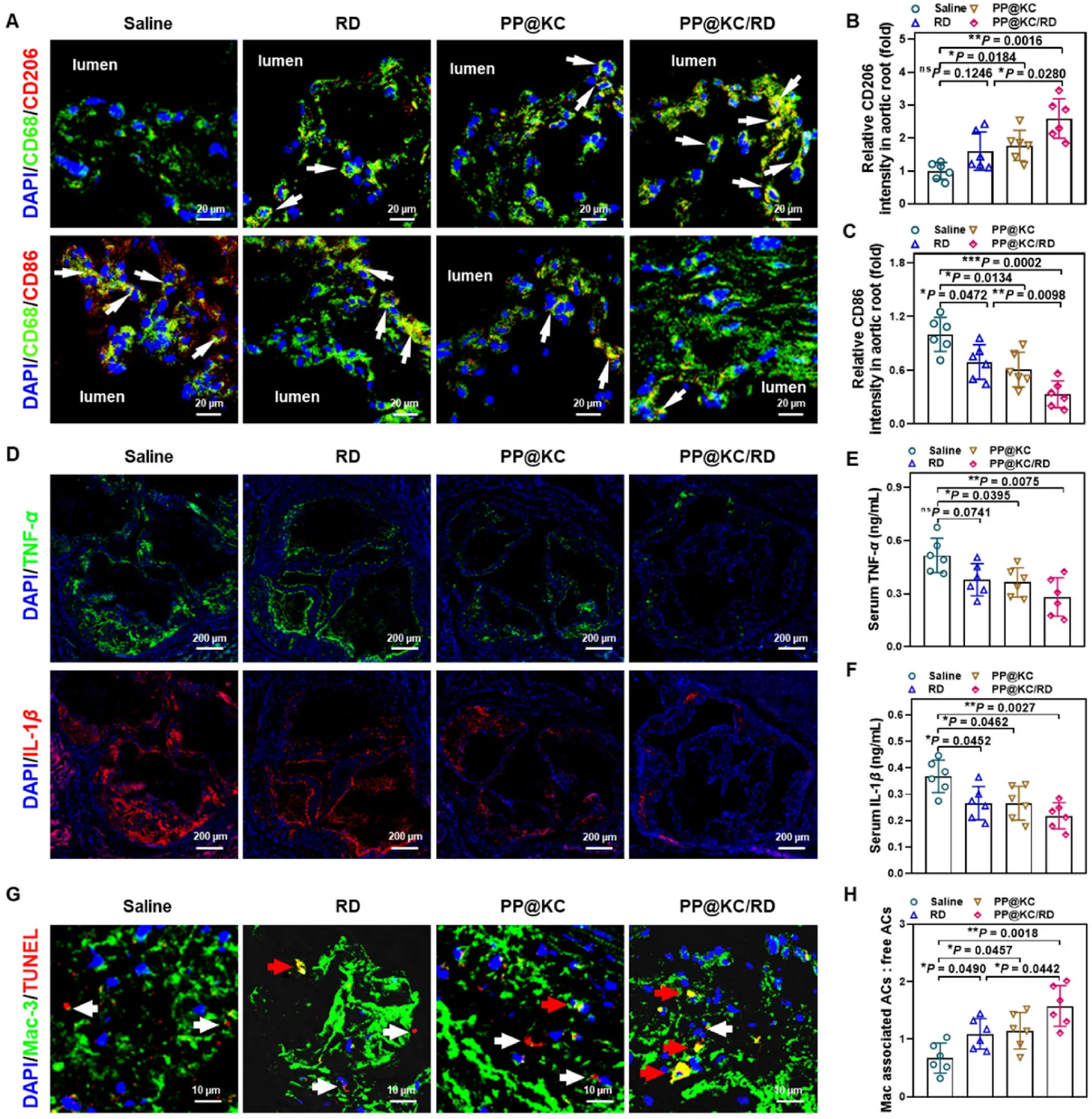
Anti-atherosclerotic mechanism of PP@KC/RD *in vivo.* (A) Representative immunofluorescence images of CD68^+^ CD206^+^ or CD68^+^ D86^+^ macrophages in cross sections of the aortic root from plaque-bearing *ApoE*^−/−^ mice after different treatments, including saline, RD, PP@KC, and PP@KC/RD. The yellow signals (indicated by white arrows) represent the overlap of green fluorescence (CD68^+^) and red fluorescence (CD206^+^ or CD86^+^). Scale bar = 20 μm. (B, C) Quantification of the fluorescence intensity ratio (*versus* saline) of CD206^+^ or CD86^+^ in the aortic roots. (D) Representative immunofluorescence images of cross sections of aortic roots illustrating levels of inflammation in the atherosclerotic plaques. Cell nuclei are stained with DAPI (blue), TNF-*α* is shown in green, and IL-1*β* is shown in red. Scale bar = 200 μm. (E, F) Levels of TNF-*α*, and IL-1*β* in serum collected from atherosclerotic mice after various treatments. (G) Representative fluorescence images of cross sections of advanced plaques showing apoptotic cells (ACs) stained with TUNEL (red), macrophages stained with Mac-3 (green), and nuclei stained with DAPI (blue). Scale bar = 10 μm. (H) Quantification of efferocytosis as the ratio of ACs (TUNEL^+^) associated with macrophages (Mac-3^+^) to free ACs (TUNEL^+^). White arrows represent free ACs, and red arrows represent ACs associated with macrophages. Data are presented as mean ± SD (*n* = 6) and analyzed using one-way ANOVA with a Dunnett T3 *post hoc* test. ∗*P* < 0.05, ∗∗*P* < 0.01, ∗∗∗*P* < 0.001, and *P* > 0.05 denotes no significance.

Given recent findings suggesting that RD treatment can enhance the efferocytic capacity in macrophages^40^, we next investigated whether lesional macrophages in PP@KC/RD-treated mice exhibited an increased ability to execute efferocytosis. To identify apoptotic cells (ACs) and macrophages, aortic root cross-sections were subjected to immunostaining using terminal deoxynucleotidyl transferase-mediated dUTP-biotin nick end labeling (TUNEL) and Mac-3, respectively^6^. Analysis of the fluorescence microscope images revealed that the ratio of macrophage-associated ACs (yellow signals indicated by red arrows, Fig. 7G) to free ACs (red signals indicated by white arrows, Fig. 7G) was significantly higher in PP@KC/RD-treated mice compared to other groups. These results demonstrate that treatment with PP@KC/RD effectively enhances the efferocytosis of lesional macrophages *in vivo* (Fig. 7H). Collectively, these findings indicate that the superior treatment efficacy of atherosclerostic plaque-targeted PP@KC/RD is primarily attributed to its ability to improve macrophage polarization and efferocytosis, resulting in reduced plaque areas and enhanced stability of advanced atherosclerotic plaques.

### 3.8 Biosafety of PP@KC/RD

To evaluate the biocompatibility of various formulations (RD, PP@KC, and PP@KC/RD), we conducted histological, blood biochemical, and hematological assays in plaque-bearing *ApoE*^−/−^ mice following 12-week treatments. First, analysis of H&E-stained organs, including the heart, liver, spleen, lung, and kidneys, revealed no obvious signs of organ injury (Supporting Information Fig. S18A). Additionally, there were no significant differences in alanine aminotransferase (ALT), aspartate aminotransferase (AST), blood urea nitrogen (BUN), and creatinine (CRE) levels between the groups (Fig. S18B). Furthermore, there were no significant differences in hematological parameters, including red blood cell count (RBC), platelet count (PLT), white blood cell count (WBC), and hemoglobin level (HGB) (Fig. S18C). Finally, body weights of the mice showed no significant changes between groups (Supporting Information Fig. S19A). These findings collectively prove that the PP@KC/RD nanotherapeutics not only exhibit superior anti-atherosclerotic efficacy by reducing plaque areas and enhancing plaque stability but also demonstrate excellent biocompatibility after long-term treatment, thereby highlighting their potential for future clinical translation.

It is worth noting that the concentrations of high-density lipoprotein (HDL), total cholesterol (TC), and low-density lipoprotein (LDL) in the serum doesn't show significant differences between the PP@KC/RD and saline groups (Fig. S19B). These findings suggest that the superior therapeutic efficacy of PP@KC/RD is primarily attributed to the anti-inflammatory properties and enhanced efferocytic capacity of lesional macrophages rather than a reduction in cholesterol levels.

## 4 Discussion

Atherosclerotic cardiovascular diseases remain the leading global cause of mortality and morbidity. Lipid-lowering therapies are emphasized as an effective strategy for reducing the risk of adverse cardiovascular events. Within this context, current guidelines recommend statins, PCSK9 inhibitors, and PCSK9 siRNA as pivotal lipid-lowering strategies for atherosclerosis patients^41^. Moreover, a range of randomized controlled trials, including the COLCOT trial^42^, LoDoCo2 trial^43^, and CANTOS trial^44^, has provided compelling evidence for the efficacy of systemic anti-inflammatory therapeutics in attenuating atherosclerotic progression. However, many of these systemic therapeutic approaches are accompanied by adverse events attributed to systemic delivery. Such unmet clinical challenges have driven an urgent need to seek alternative strategies for the targeted delivery of therapeutic agents. These strategies are expected to offer targeted inhibition of the intrinsic, disease-causing pathways while simultaneously mitigating adverse effects compared to systemic administration.

In addition to lipid-lowering and anti-inflammatory therapies, genetic and mechanistic studies have underscored the pivotal role of defective efferocytosis in driving atherosclerotic plaque expansion^33^. Pre-clinical studies have yielded promising results using nanomedicine-based targeted delivery strategies to facilitate pro-efferocytosis therapy in atherosclerotic cardiovascular diseases^4^^,^^45^. For example, an siRNA nanoparticle conjugated with a macrophage-targeting ligand, S2P, has been developed to effectively silence *Camk2g*, a plaque-destabilizing macrophage molecule involved in impaired efferocytosis. The application of this nanoplatform on atherosclerotic mice effectively enhanced macrophage efferocytosis, reduced the necrotic core area, and increased plaque stability^6^. Additionally, Flores and colleagues designed a single-walled carbon nanotubes loaded with inhibitors of the CD47–SIRP*α* signaling axis^37^. This nanosystem demonstrated a remarkable ability to accumulate within atherosclerotic plaques, enhance lesional phagocytosis, and reduce plaque burden in atherosclerotic mice, thereby highlighting the promise of nanoparticle-mediated macrophage efferocytosis restoration in atherosclerosis treatment.

In our recent study, we demonstrated the antioxidative role of PP in scavenging ROS to reduce the oxidative stress in atherosclerosis plaques^17^. Additionally, this nanoformulation was found to decrease the expression of pro-inflammatory cytokines, thereby resolving local inflammation and contributing to the treatment of atherosclerosis. Building on these pioneering findings, we further discovered that PP extend their function beyond anti-oxidation by enhancing macrophage efferocytosis. This enhancement is achieved through upregulation of the pro-efferocytosis receptor MerTK and downregulation of CaMKII*γ*, highlighting the novel pleiotropic effects of PP in reducing atherosclerotic burden in mice. Specifically, we have developed an atherosclerotic plaque targeting nanoplatform, PP@KC, as a carrier for the delivery of RD biological drug to effectively treat atherosclerosis. The KC11 functionalization step is modular and uses readily available materials, and it can be integrated after nanoparticle formation without changing the core formulation process (self-assembly/adsorption step under mild conditions). KC11 enables ligand-mediated, cell-selective targeting to lesional macrophages, thereby overcoming the limited specificity inherent to passive delivery. This receptor-associated targeting mechanism markedly. Moreover, strong literature precedent collectively indicate that the electrostatic KC11-PEG coating is sufficiently stable *in vivo* to enable effective plaque targeting^46^^,^^47^. This plaque targeted drug delivery nanoplatform demonstrates superiority in promoting efferocytosis, enhancing the M2-like macrophage phenotype, and exerting anti-inflammatory effects, while also exhibiting good biocompatibility. By binding with overexpressed Col IV in atherosclerotic plaques *via* the KC11 targeting ligand, PP@KC/RD efficiently accumulates within atherosclerotic plaques. Subsequently, RD is continuously released from PP@KC/RD, thereby increasing the local RD concentration. Compelling evidence from both *in vitro* and *in vivo* experiments confirms that the combination of RD and PP@KC significantly attenuates the progression of atherosclerosis. Mechanistic studies show that PP@KC/RD facilitate the polarization of M2-like macrophages by effectively up-regulating the PPAR-*γ* expression, which enhances efferocytosis and resolves inflammation^31^^,^^32^, thereby contributing to the reduction in plaque area and the stabilization of plaques. While the prior study focused on RD delivery using black phosphorus mainly as an antioxidative carrier, the present work emphasizes the synergistic integration of three functions: (i) phosphorene-mediated immunomodulation, (ii) KC11-enabled precision delivery to lesional macrophages, and (iii) locally concentrated RD signaling. The data in Fig. 6 show that this integrated PP@KC/RD construct produces significantly greater plaque regression and inflammatory resolution than RD or PP alone, indicating a synergistic rather than additive therapeutic effect. Beyond serving as a delivery scaffold, phosphorene is shown here to exert previously unrecognized pleiotropic effects on macrophage efferocytosis and M2 polarization, which were not explored in the prior report. PP@KC/RD integrates advantages in targeting logic, efferocytosis-enhancing potency, scalable manufacturing, and regulatory compatibility, thereby offering a practical and translatable pathway toward IND-enabling studies and potential first-in-human evaluation. However, we also acknowledge that our current work is limited to small-animal studies, which, while informative for mechanistic investigation, only partially recapitulate the anatomical, physiological, and immunological features of human atherosclerosis. To address this gap, future studies using large-animal models—such as pig models of experimentally induced atherosclerosis—will be essential for evaluating the *in vivo* therapeutic efficacy, safety, and biodistribution of this nano-immunotherapy^48^, ^49^, ^50^.

In summary, our study introduces a PP and RD biologics-based nano-immunotherapeutic approach for treating atherosclerosis by restoring efferocytosis and improving the M2-like polarization of lesional macrophages. This nanoplatform shows potential as an engineered biological nanomedicine for the treatment of atherosclerosis and other diseases associated with impaired efferocytosis.

## Author contributions

Zhongshan He: Writing – review & editing, Writing – original draft, Visualization, Validation, Software, Methodology, Investigation, Formal analysis, Data curation. Yaoyao Luo: Visualization, Validation, Software, Methodology, Investigation. Yongjiang Li, Yingjun Fan, Yi Zhang, Shengbin Liu, Chengjian Cao, and Zhuoming Zhou: Methodology, Investigation. Haixing Shi, Mengran Guo, and Yuting Chen: Visualization. Yupei Zhang, and Duotian Qin: Methodology. Wei Chen: Writing – review & editing, Supervision, Resources, Project administration. Zhongshan He and Xiangrong Song: Writing – review & editing, Supervision, Funding acquisition, Conceptualization.

## Conflicts of interest

The authors declare no conflicts of interest.

## Appendix A Supporting information

The following is the Supporting Information to this article.
