# Supplementary material for: Remodeling macrophage efferocytosis and phenotype via targeted nanoimmunotherapy for atherosclerosis
Source: Acta Pharm Sin B. 2026 Aug 9;16(10):6984–7000. doi: 10.1016/j.apsb.2026.08.006 (PMC13712345; doi:10.1016/j.apsb.2026.08.006)
Supplement: Supplementary file 1 [file mmc1.pdf]

Supporting Information for

ORIGINAL ARTICLE

Remodeling macrophage efferocytosis and phenotype *via* targeted nanoimmunotherapy for atherosclerosis

Zhongshan He[a,d,†,*], Yaoyao Luo[b,†], Yongjiang Li[c],Shuping Yang[a], Yingjun Fan[a], Yi Zhang[a], Shengbin Liu[a], Zhuoming Zhou[c], Chengjian Cao[a], Haixing Shi[a], Mengran Guo[a], Yuting Chen[a], Yupei Zhang[a], Duotian Qin[c], Wei Chen[c,*], Xiangrong Song[a,*]

[a]*Department of Critical Care Medicine, Department of Clinical Pharmacy, Frontiers Science Center for Disease-Related Molecular Network, State Key Laboratory of Biotherapy and Cancer Center, West China Hospital, Sichuan University, Tianfu Jincheng Laboratory, Chengdu 610041, China*

[b]*Laboratory of Cardiac Structure and Function, Institute of Cardiovascular Diseases, West China Hospital, Sichuan University, Chengdu 610041, China*

[c]*Center for Nanomedicine and Department of Anesthesiology, Brigham and Women's Hospital, Harvard Medical School, Boston, MA 02115, USA*

[d]*Department of Bioengineering, School of Engineering and Applied Science, University of Pennsylvania, Philadelphia, PA 19104, USA*

Received 27 October 2025; received in revised form 8 February 2026; accepted 25 February 2026

*Correspondence authors.

E-mail addresses: songxr@scu.edu.cn (Xiangrong Song), wchen45@bwh.harvard.edu (Wei Chen), zhongshan_he@163.com (Zhongshan He).

[†]These authors made equal contributions to this work.

Table of contents

Supplementary Experimental Procedures……………………………………………….. 3

Supplementary Figure…………………………………………………………..…………12

**Figure S1**	Synthesis and characterization of KC11-PEG2k-NH_2
**Figure S2**	Characterization of PP@KC/RD and RD release studies
**Figure S3**	Parallel-plate flow-chamber evaluation of shear-dependent RD release from PP@KC/RD
**Figure S4**	Cellular uptake analysis of PP@KC/FITC in LPS-stimulated RAW264.7 cells
**Figure S5**	Cellular uptake analysis of PP@KC/FITC in ox-LDL-stimulated RAW264.7 cells
**Figure S6**	Cellular uptake analysis of PP@FITC in RAW264.7 cells
**Figure S7**	Gating strategy used in the flow cytometry evaluation of M2-like macrophage polarization
**Figure S8**	Investigation of RAW264.7 cells efferocytosis efficiency
**Figure S9**	Expression of macrophage inflammation-resolving pathway genes
**Figure S10**	*In vivo* pharmacokinetics and biodistribution evaluation
**Figure S11**	Vascular cell specificity of PP@KC/RD/Cy5 using flow cytometry analysis
**Figure S12**	CLSM images showed accumulation of PP@KC/Cy7 in aortic roots
**Figure S13**	Splenic cell specificity of PP@KC/RD/Cy5 using flow cytometry analysis
**Figure S14**	Blood cell specificity of PP@KC/RD/Cy5 using flow cytometry analysis
**Figure S15**	Recruitment of macrophages and Ly-$6C^{hi}$ cells change
**Figure S16**	PP@KC/RD therapy reduced lesion areas and improved plaque stability
**Figure S17**	PP@KC/RD therapy decreased TNF-α and IL-1β expression in aortic root sections
**Figure S18**	Safety evaluation of plaque-bearing *ApoE*$^{-/-}$ mice after receaving various treatments
**Figure S19**	Analysis of body weight and blood lipid of *ApoE*$^{-/-}$ mice after receaving various treatments

Supplementary Table…………………………………………………………..………..…29

**Table S1**	Primer sequences used for the RT-qPCR analysis of RAW264.7 cells
**Table S2**	Antibodies used for the flow cytometry analysis of aortic cells
**Table S3**	Antibodies used for the flow cytometry analysis of splenic cells
**Table S4**	Antibodies used for the flow cytometry analysis of blood cells

Supplementary Reference…………………………………………………………...…..…30

1. Supplementary Experimental Procedures

1.1. Synthesis of phosphorene (PP) nanosheets

The phosphorene (PP) nanosheets were prepared based on a liquid-phase exfoliation strategy in an aqueous solution. Briefly, 45 mg of bulk black phosphorous crystal was added to a 50 mL of ultrapure water followed by N_2 bubbling for 60 min to remove the dissolved oxygen. The mixture was sonicated for 12 h under an ice water bath by an ultrasound probe (Scientz-IID, 500W, 50% power, on/off cycle: 20/5 s, Ningbo Scientz Biotechnology Co., Ltd., Ningbo, China). To remove the unexfoliated bulk PP, the solution then was centrifuged at 300 × *g* for 10 min. Afterward, the collected supernatant was centrifuged at 7,000 × *g* for 10 min to obtain the PP nanosheet pellets.

1.2. Synthesis of KC11-PEG-NH_2

KC11-PEG-NH_2 (Mw: 3,350 Da) was synthesized using a two-step reaction approach. Briefly, KC11-SH (Mw: 1,150 Da, 100 mg, 0.0865 mmol) was combined with Mal-PEG-NH_2 (Mw: 2,200 Da, 173.0 mg, 0.0786 mmol) in 5 mL of anhydrous dimethylformamide (DMF) in a nitrogen atmosphere. The mixture was under continuous stirring at room temperature for 24 h. After the reaction, both the DMF and residual KC11-SH were removed by dialysis (MWCO = 2000 Da). Finally, to remove any remaining moisture, the product was subjected to lyophilization, resulting in the KC11-PEG-NH_2 product.

1.3. Preparation of PP@KC and PP@KC/RD

Briefly, 10 mg of KC11-PEG-NH_2 was added to a 10 mL water solution of PP (50 μg/mL), and the resulting mixture was sonicated for 10 min at room temperature. Then, the mixture was stirred for an additional 4 h in a dark environment. Finally, the excess KC11-PEG-NH_2 was removed by centrifugation and the KC11-PEG-NH_2-coated PP (PP@KC) were further washed with water twice.

For RD loading, a water solution of PP@KC (50 μg/mL was mixed with different concentrations of RD (25, 50, 75, 100, or 125 μg/mL). The mixture was then stirred for 12 h in a dark environment to allow for RD loading onto the RD@KC. Following the incubation period, the RD-loaded PP@KC, designated as PP@KC/RD, was collected by centrifugation (9,000 *g*, 50 min) at 4 °C. The collected PP@KC/RD was then washed twice with water. Afterward, PP@KC/RD were re-suspended in water and stored at 4 °C for future use.

The calculation of RD loading capacity on PP@KC/RD was determined using the formula: (mass of loaded RD/mass of PP@KC) × 100%. To determine the amount of loaded RD, the concentration of unloaded RD in the supernatant collected during each wash was measured using

high performance liquid chromatography, which of parameters were as follows: chromatographic column: Waters® Symmetry 250 × 4.6 mm, 4 μm; mobile phase: 0.1% acetic acid aqueous solution: methanol (25/75, *v*/*v*); flow rate: 0.5 mL/min; detection wavelength: 302.2 nm; column temperature: 30 °C; injection volume: 20 μL. The loading capacity was calculated as following Eq. (1)

$$\text{Loading capacity (\%)} = \frac{Mi - Mn}{Mt} \times 100\% \qquad (1)$$

Where M_n is the mass of free RD, and M_i and M_t are the initial mass of RD and PP@KC at different feeding ratios, respectively. The experiments were conducted in triplicate to ensure the reproducibility of the results.

The surface modification of PP with fluorescent PEG, such as FITC-PEG-NH_2 and Cy7-PEG-NH_2, was prepared using the same strategy. The resulting nanomaterials were denoted as PP@FITC and PP@Cy7, respectively. The entire process was executed in a dark environment to prevent the bleaching of FITC and Cy7.

1.4. Characterization of PP-based NSs

The morphology of PP and PP@KC was characterized using a transmission electron microscope (TEM) (HITACHI, HT7700, 100 kV, Hitachi, Tokyo, Japan). The thickness of PP and PP@KC was measured using an atomic force microscope (AFM) (Bruker, Santa Barbara, USA). The concentration of PP was determined using an inductively coupled plasma atomic emission spectroscopy (ICP-AES) ((IRIS Intrepid II XSP, thermo Electron Corporation, Waltham, USA). The hydrodynamic size distribution and zeta potential values of PP and PP@KC were measured using a Malvern Zetasizer Nano-ZS instrument (ZEN3600, Malvern Instruments, Malvern, UK). X-ray diffraction pattern was obtained using a Bruker D8 Discover XRD system. Raman scattering spectra were acquired using a high-resolution confocal Raman microscope (Horiba Jobin Yvon Lab Ram HR VIS, excitation wavelength: 633 nm). All experiments were conducted in triplicate to ensure the reproducibility of the results.

1.5. H_2O_2 scavenging capability

The H_2O_2 scavenging capability of PP and PP@KC was assessed using the hydrogen peroxide assay kit (Nanjing Jiancheng, Jiangshu, China). Various concentrations of PP and PP@KC (10 μg/mL, 20 μg/mL, 40 μg/mL, 80 μg/mL, and 160 μg/mL) were incubated with 50 mmol/L H_2O_2 at 37 °C for 6 h. Following the incubation, the remaining H_2O_2 reacted with ammonium molybdate to form a stable yellow complex, which exhibited a maximum absorbance at 405 nm.

The concentration of H_2O_2 and the H_2O_2 scavenging efficiency were subsequently calculated according to the manufacturer's instructions.

1.6. Degradation of PP@KC in PBS with H_2O_2

The degradation of PP@KC (50 µg/mL) was conducted in PBS solutions containing 0, 0.1, 0.5, or 1.0 mmol/L H_2O_2 at various time intervals. The absorbance of the PP@KC solution was measured using a UV–Vis–NIR spectrophotometer (U-3900, Hitachi High-Tech Corporation, Tokyo, Japan), and the degree of degradation was calculated based on the difference in absorbance using the following Eq. (2):

$$\text{Degradation degree (\%)} = (1 - I_x/I_0) \times 100 \qquad (2)$$

where I_x is the absorbance at 808 nm in the UV–Vis–NIR spectrum at the specific time points and I_0 is the initial absorbance of the solution.

1.7. Release of RD from PP@KC/RD

The evaluation of RD release from ROS-responsive PP@KC/RD was performed in a PBS solution with or without H_2O_2 (1 mmol/L). In general, a 2 mL of solution of PP@KC/RD (with RD/PP@KC feeding ratio of 2) was added into a dialysis bag (MWCO = 14,000 Da) which was subsequently immersed in a 10 mL of buffer solution. At different time intervals, 1 mL of the incubation medium was collected to determine the concentration of RD using HPLC. Simultaneously, 1 mL of fresh medium was added back into the buffer solution to maintain a constant volume. The cumulative release of RD from ROS-responsive PP@KC/RD was determined using the following Eq. (3):

$$M_n = C_n \times V + \sum_{i=1}^{n-1} C_i \times V_i \quad \text{Cumulative drug release (\%)} = \frac{M\text{n}}{M} \times 100\% \qquad (3)$$

where M_n is the cumulative amount of released drug at time n, C_n is the concentration of the released drug at time n, V is the total volume of dissolve medium (10 mL), C_i is the concentration of RD measured at time point i, V_i is the collected volume from dissolve medium at time point i (1 mL), and M is the initial amount of drug in PP@KC. The RD release experiment was conducted in triplicate.

1.8. Shear-responsive drug release from PP@KC/RD

In vitro microfluidic model was constructed to reproduce the shear-stress microenvironment experienced by atherosclerotic plaques and to evaluate drug-release under these conditions. The PP@KC/RD PBS suspension (pH 7.4) was continuously recirculated through stenotic channels

whose wall shear stress (WSS) was precisely controlled by connecting the customised ibidi Pump System to microfluidic chips calibrated for oscillatory shear stress (OSS, 0.5 dyn cm^{-2}), low laminar shear stress (LSS, 12 dyn cm^{-2}) and high shear stress (HSS, 30 dyn cm^{-2})[1–4]. Release was compared with the conventional static condition (0.5 dyn cm^{-2}) by perfusing H_2O_2-containing PBS (1 mmol/L) and collecting downstream samples for HPLC quantification of RD. At predetermined intervals (0, 0.5, 1, 4, 8, 12 and 24 h) aliquots of the circulating medium were withdrawn, ultrafiltered (10 kDa MWCO) to isolate free RD, and quantified by HPLC. The cumulative release of RD from PP@KC/RD was determined using the same equation as below.

1.9. Cell culture

RAW264.7 (mouse macrophage) and HUVEC (human umbilical vein endothelium) cell lines were purchased from ATCC. All cell lines were cultured in T-75 flasks with vented caps in DMEM supplemented with 10% FBS and 1% antibiotics (100 Units/mL penicillin and 100 μg/mL streptomycin) in a humidity-controlled incubator at 37 °C with 5% CO_2. The culture medium was replaced daily, and the cells were harvested by trypsinization with 0.25% trypsin-EDTA for passaging every other day.

1.10. Cellular uptake

The uptake of different nanoparticle formulations was examined in lipopolysaccharide (LPS)-treated RAW264.7 cells (inflammatory macrophages) and oxidized low-density lipoprotein (ox-LDL)-treated RAW264.7 cells (macrophages-derived foam cells) using confocal laser scanning microscopy (CLSM) and flow cytometry analysis. The cells were seeded in cell culture dishes with a glass bottom (NEST Biotechnology, Wuxi, China) at a density of 1×10^5 cells per dish. After 24 h of attachment at 37 °C, the culture medium of the cells was replaced with fresh medium containing PP@FITC or PP@KC/FITC, and the cells were incubated further at 37 °C for 1, 2, 3, or 4 h. After the incubation, the cells were washed three times with PBS, and then stained with Hoechst for 15 min. Afterward, the cells were washed with PBS three times before observation using a CLSM (LSM880, Carl Zeiss, Oberkochen, Germany).

For flow cytometry experiments, cells were treated similarly. The cells were then suspended in PBS and analyzed using a fluorescence-activated cell sorting system at an excitation wavelength of 488 nm (FACS, AccuriC6, BD Biosciences, Franklin Lakes, NJ, USA). The results were analyzed using FlowJo software and GraphPad Prism 8.

1.11. In vitro biocompatibility assay

The viabilities of RAW264.7 and HUVECs following various treatments were assessed using an MTT assay. Briefly, the cells were seeded in 96-well plates at a density of 1×10^5 cells per mL and allowed to attach for 24 h. Subsequently, the culture medium was removed, and then added to 100 μL of fresh medium containing PP@KC at different concentrations of PP (0, 10, 50, 200, and 400 μg/mL). After an additional 24 h incubation, the medium was replaced with 40 μL of DMEM containing the MTT assay reagent. The absorbance of the medium at 550 nm was measured using a microplate reader (Model 680, Bio-Rad, Hercules, CA, USA) to determine cell viability.

1.12. Investigation of macrophage phenotype in vitro

To investigate the macrophage phenotype *in vitro*, RAW264.7 cells were initially stimulated with LPS (500 ng/mL) and subsequently incubated with different formulations, including RD (10 nmol/L), PP@KC (50 μg/mL), or PP@KC/RD (50 μg PP@KC/RD per mL; 10 nmol/L RD) for 24 h. Untreated cells served as control group. Following treatment, the cells were treated with FC Block (Cat. No. 553141, BD Biosciences), followed by incubation with FITC rat anti-mouse F4/80 (157310, BioLegend, 3 μg/mL) and APC rat anti-mouse CD86 (Cat. No. 558703, BD Biosciences, 2 μg/mL) at 4 °C for 30 min. Subsequently, the cells were washed, fixed with Fix/Perm buffer (BD Biosciences), and incubated with PE anti-mouse CD206 (MMR) antibody (Cat. No. 141706, BioLegend, 2 μg/mL) at 4 °C for 30 min. The expression of CD86 and CD206 on the macrophages was measured by flow cytometry (BD Biosciences San Jose, CA, USA), and the data were analyzed using FlowJo software.

For immunofluorescence staining, the treated cells were fixed in 4% paraformaldehyde for 15 min and then permeabilized with PBS containing 2% Triton X-100 for 10 min. Afterward, the cells were washed three times with ice-cold PBS and then blocked with a solution of 0.5% bovine serum albumin (BSA) and 10% normal goat serum in PBS for 40 min. Following blocking, the cells were incubated overnight at 4 °C with CD206 (Abcam, Cat. No. ab64693, rabbit, 1 μg/mL), and CD86 (Abcam, Cat. No. ab119857, rat, 1 μg/mL) primary antibodies. Subsequently, the cells were washed with PBS three times, and then incubated with the corresponding Alexa fluor 488-conjugated goat anti-rabbit IgG (CST, Cat. No. 4412, 2 μg/mL) and Alexa fluor 594-conjugated goat anti-rat IgG (Abcam, Cat. No. ab150160, 2 μg/mL) in the dark at room temperature for 2 h. Cell nuclei were stained with DAPI. Finally, the immunostained images were captured using a CLSM (LSM880, Carl Zeiss, Oberkochen, Germany), and Image-Pro Plus 6.0 software was performed to quantify the fluorescence signal intensities of the captured images.

1.13. In vitro anti-inflammatory capability of PP@KC/RD

To investigate the anti-inflammatory capability of PP@KC/RD *in vitro*, RAW264.7 cells were seeded in 96-well plates at a density of 1.0 $\times 10^4$ cells per well and allowed to attach for 24 h. After attachment, the medium was removed, and then added to fresh medium containing RD (10 nmol/L), PP@KC (50 μg/mL), or PP@KC/RD (50 μg PP@KC/RD per mL; 10 nmol/L RD) and incubated for 2 h. Then, the cells were treated with lipopolysaccharide (LPS, 500 ng/mL) for 12 h. Finally, the supernatants were collected, and the concentrations of various cytokines (TNF-α, IL-6, IL-1β, and IL-10) were determined using an ELISA assay (Solarbio, Shanghai, China). Cells without any treatment and cells treated with LPS only served as the control and negative control (model), respectively.

1.14. In vitro efferocytosis assay of PP@KC/RD

To investigate the phagocytic capability of macrophages *in vitro*, live RAW264.7 macrophages were co-incubated with apoptotic RAW264.7 macrophages. To obtain the fluorescent apoptotic cells, RAW264.7 cells were treated with CellTracker Red (2 μmol/L; Life Technologies) and then incubated with TNF-α (50 ng/mL; R&D) for 24 h in a dark environment. Meanwhile, the live RAW264.7 macrophages were pre-incubated with various formulations, including RD (10 nmol/L), PP@KC (50 μg/mL), and PP@KC/RD (50 μg of PP@KC/RD per mL; 10 nmol/L RD) for 2 h. Subsequently, the live macrophages were labeled with CellTracker Green (1.5 μmol/L; Life Technologies) for 30 min in a dark environment. Cells without any treatment and cells treated with LPS only served as the control and negative control (model), respectively. The live RAW264.7 macrophages and apoptotic RAW264.7 cells were then co-cultured in 24-well plates at a density of 1×10^5 and 3×10^5 cells per well, respectively, in serum-free medium. After a 2-h co-incubation, the cells were washed with medium three times, and fluorescence microscope images were acquired using CLSM. The phagocytic activity was evaluated by determining the percentage of macrophages that had engulfed at least one apoptotic cell (efferocytosis index).

1.15. Western blotting assay

The treated RAW264.7 cells first were harvested and washed with cold PBS. Proteins were lysed and extracted in RIPA lysis buffer, and then separated by 10% SDS-PAGE. Afterwards, proteins of samples were transferred to polyvinylidene difluoride membranes (Millipore) using a wet transfer device. Subsequently, the membranes were blocked in a tris-buffered saline containing tween 20 and 5% BSA at room temperature. After 1 h of blocking, the membranes were incubated overnight at 4 °C with primary antibodies, including PPAR-γ (Cell Signaling Technology, Cat.

No. 2435T, 1/1000, *v*/*v*), CaMKIIγ (Cell Signaling Technology, Cat. No. 25276S, 1/1000, *v*/*v*), MerTK (Cell Signaling Technology, Cat. No. 38102S, 1/1000, *v*/*v*), and β-actin (horseradish peroxidase (HRP) conjugate, Cell Signaling Technologies, Cat. No. 5125, 1/5000, *v*/*v*). Subsequently, the membranes were washed with TBST three times, and shakily incubated with HRP-conjugated secondary antibodies at room temperature for 2 h. The images of samples were acquired using an enhanced chemiluminescence kit (Millipore Corporation, Billerica, MA, USA), and the quantification of signals was performed using Image-Pro Plus 6.0 software.

1.16. Quantitative real-time PCR assay

Total RNA was extracted from the treated RAW264.7 cells using the TRIzol reagent (Takara) according to the manufacturer's instruction. Subsequently, complementary DNA was synthesized using a PrimeScript RT reagent kit (Takara). Quantitative real-time PCR (RT-qPCR) analysis was conducted on a detection system (CFX96, Bio-Rad, Hercules, CA, USA). The primer sequences used in this study are listed in Table S1.

1.17. In vivo pharmacokinetics and biodistribution evaluation

To evaluate the *in vivo* pharmacokinetic characteristics of Cy5-labeled nanomedicine, we employed a combined approach involving small-animal *in vivo* imaging and quantitative *ex vivo* organ analysis. Atherosclerotic plaque-bearing *ApoE*$^{-/-}$ mice served as experimental subjects and were injected with Cy5-labeled nanomedicine *via* the tail vein at a dose of 10 μg of PP@KC preloaded with 100 ng of RD per mouse. Blood samples were collected *via* retro-orbital bleeding at 0.5, 1, 2, 4, 8, 12, 24, 48, and 96 h post-administration (n = 3 per time point). Samples were anticoagulated with heparin and centrifuged at 1,000 *g* for 10 min to separate plasma. Cy5 fluorescence intensity in plasma was measured using a multimode microplate reader at excitation/emission wavelengths of 640 nm/680 nm and converted to Cy5 concentration based on a standard curve. Simultaneously, mice were euthanized at each time point, and major organs including the heart, liver, spleen, lungs, and kidneys were rapidly harvested, rinsed with PBS, blotted dry with filter paper, and weighed. The organs were then subjected to *ex vivo* fluorescence imaging using the IVIS Spectrum small-animal *in vivo* imaging system (excitation/emission wavelengths 640 nm/680 nm, exposure time 1–5 s). Regions of interest (ROIs) were delineated using Living Image software to calculate fluorescence intensity per unit tissue mass, and ultimately converted to organ Cy5 concentration (μmol/L) to assess the whole-body spatiotemporal distribution profile and major targeting organs of the nanomedicine.

1.18. Recruitment of macrophages and Ly-6C^{hi} cells change

Twelve-week HFD-fed *ApoE*$^{-/-}$ mice were randomly distributed into three groups (n = 5 per group), and intravenously administered saline and PP@KC/RD/Cy5 (100 ng of RD/Cy5 per mouse) twice with an interval of 3 days. Following the intravenous administration, biodistribution studies were conducted. The whole aorta and peripheral blood of treated *ApoE*$^{-/-}$ mice were quickly harvested, homogenized, and digested to prepare single-cell suspensions for flow cytometry staining analysis. The red blood cells (RBCs) were removed from peripheral blood by incubation with ammonium-chloride-potassium lysis buffer for 10 minutes on ice. The aortas were homogenized and digested using a mixture of enzymes, including collagenase I (450 U/mL; Cat. No. C0130, Sigma–Aldrich), collagenase XI (125 U/mL; Cat. No. C7657, Sigma–Aldrich), hyaluronidase (60 U/mL; Cat. No. H3506, Sigma–Aldrich), and deoxyribonuclease I (60 U/mL; Cat. No. D4527, Sigma–Aldrich) in Hank's balanced salt solution at 37°C for 1 h. Digested tissue was passed through a 70 μm strainer to obtain single-cell suspensions. Subsequently, cell suspensions were stained with the Zombie Red™ Fixable Viability Kit (Cat. No. 423110, BioLegend) in the dark at room temperature for 15 min to distinguish between viable and dead cells. Afterwards, cell populations were detected using the LSR II flow cytometry system (FACS, AccuriC6, BD Biosciences, Franklin Lakes, NJ, USA) and analysed using FlowJo v10.0.7. Specific analysis involved sequential gating for non-debris (forward scatter area versus side scatter area), singlets (forward scatter area versus forward scatter height), and viable cells (PETexas Red negative). The viable, single cells were then analyzed for Cy5 average fluorescence intensity, or Cy5 positivity to determine the percentage of Cy5^{+} cells in each sample. To assess vascular cell specificity, single-cell suspensions from the aorta and blood were counted and diluted to 1 × 10^{6} cells per 100 μL. After incubation with the Zombie Red™ Fixable Viability Kit (Cat. No.: 423110, BioLegend) in the dark at room temperature for 15 min, cells were washed with FACS buffer (Dulbecco's PBS supplemented with 2% FBS, 0.5% bovine serum albumin, and 0.05% NaN_3). Subsequently, the cells were treated with FC Block (Cat. No. 553141, BD Biosciences), followed by staining with various antibodies for 45 minutes at 4 °C: PerCP-Cy5.5-anti-CD45 (clone 30-F11; Cat. No. 550994, BD Biosciences); FITC-antiCD90.2 (clone 53-2.1; Cat. No. 561973, BD Biosciences); FITC-anti-CD45R (clone RA3-6B2; Cat. No. 553088, BD Biosciences); FITC-anti-CD49b (clone DX5; Cat. No. 553857, BD Biosciences); FITC-anti-NK1.1 (clone PK136; Cat. No. 553164, BD Biosciences); FITC-anti-Ter119 (clone TER119; Cat. No. 116206, BioLegend); FITC-anti-Ly6G (clone 1A8; Cat. No. 127606, BioLegend); PE-Cy7-anti-CD11b (clone M1/70; Cat. No. 552850, BD Biosciences); BV421-anti-F4/80 (clone BM8; Cat. No. 565411, BD Biosciences); BV605-Ly-6C (clone AL-21; Cat.

No. 563011, BD Biosciences). Cell suspensions were subjected to LSR II flow cytometry FACS, AccuriC6, BD Biosciences, Franklin Lakes, NJ, USA) and analysed using FlowJo v10.0.7. Macrophages were identified as $CD45^{+}CD11b^{hi}Lin^{-/low}F4/80^{hi}$ cells, $Ly6C^{hi}$ monocytes were identified as $CD45^{+}CD11b^{hi}Lin^{-/low}Ly\text{-}6C^{hi}$ cells.

1.19. Quantitation of pro-inflammatory cytokine in the serum

The samples of blood were collected from *ApoE*$^{-/-}$ mice treated for 12 weeks. The serum concentrations of pro-inflammatory cytokines, such as TNF-α, and IL-1β, were measured using ELISA kits, according to the manustructure's instructions.

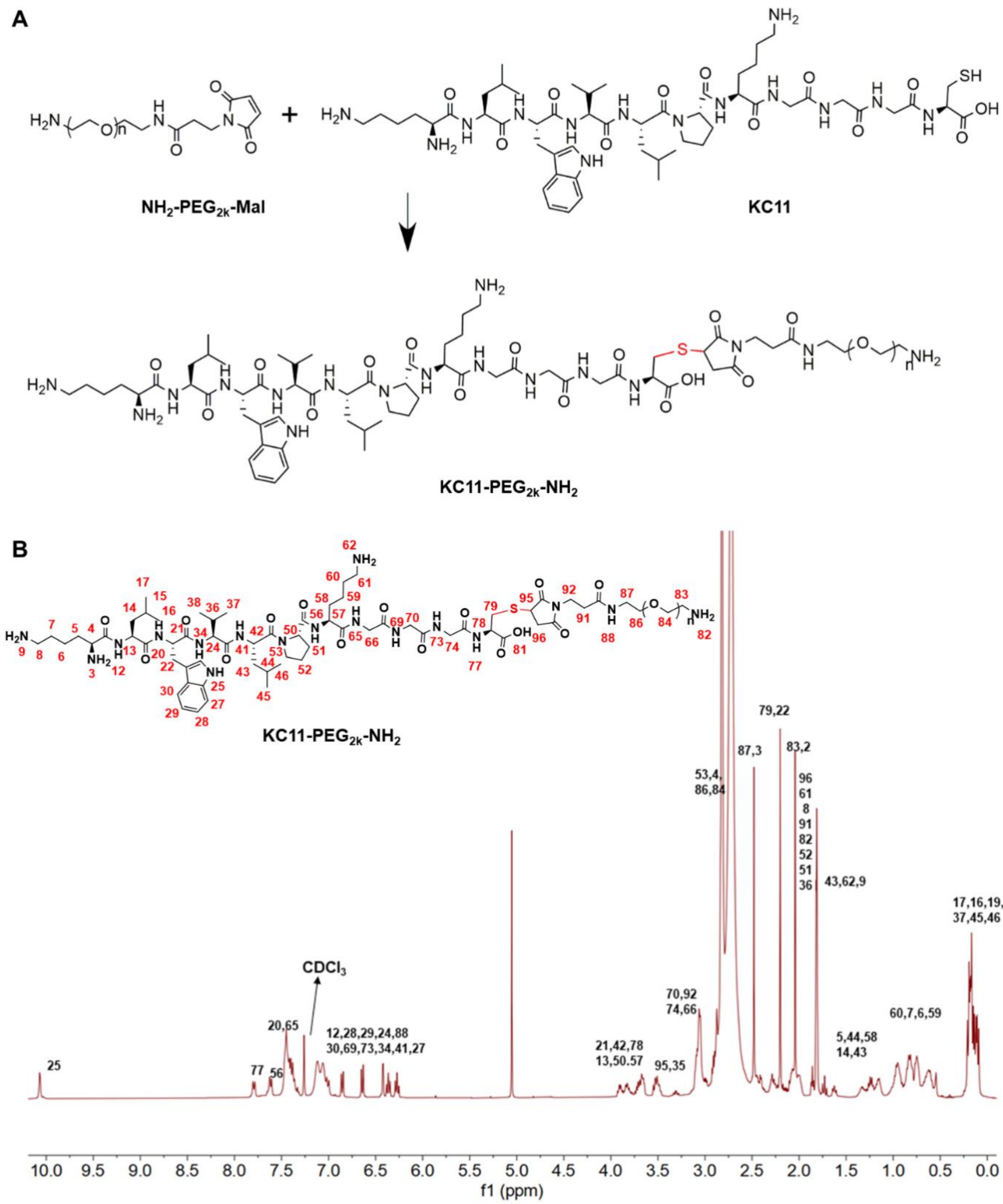

Figure S1 Synthesis and characterization of KC11-PEG2k-NH_2. (A) Synthesis procedure and (B) nuclear magnetic resonance hydrogen spectrum (^1H NMR) of KC11-PEG2k-NH_2. KC11-PEG2k-NH_2 was synthesized through a thioester bond formation reaction between the KC11 peptide (KLWVLPKGGGC-SH) and heterobifunctional maleimide-polyethylene glycol-amine (Mal-PEG-NH_2).

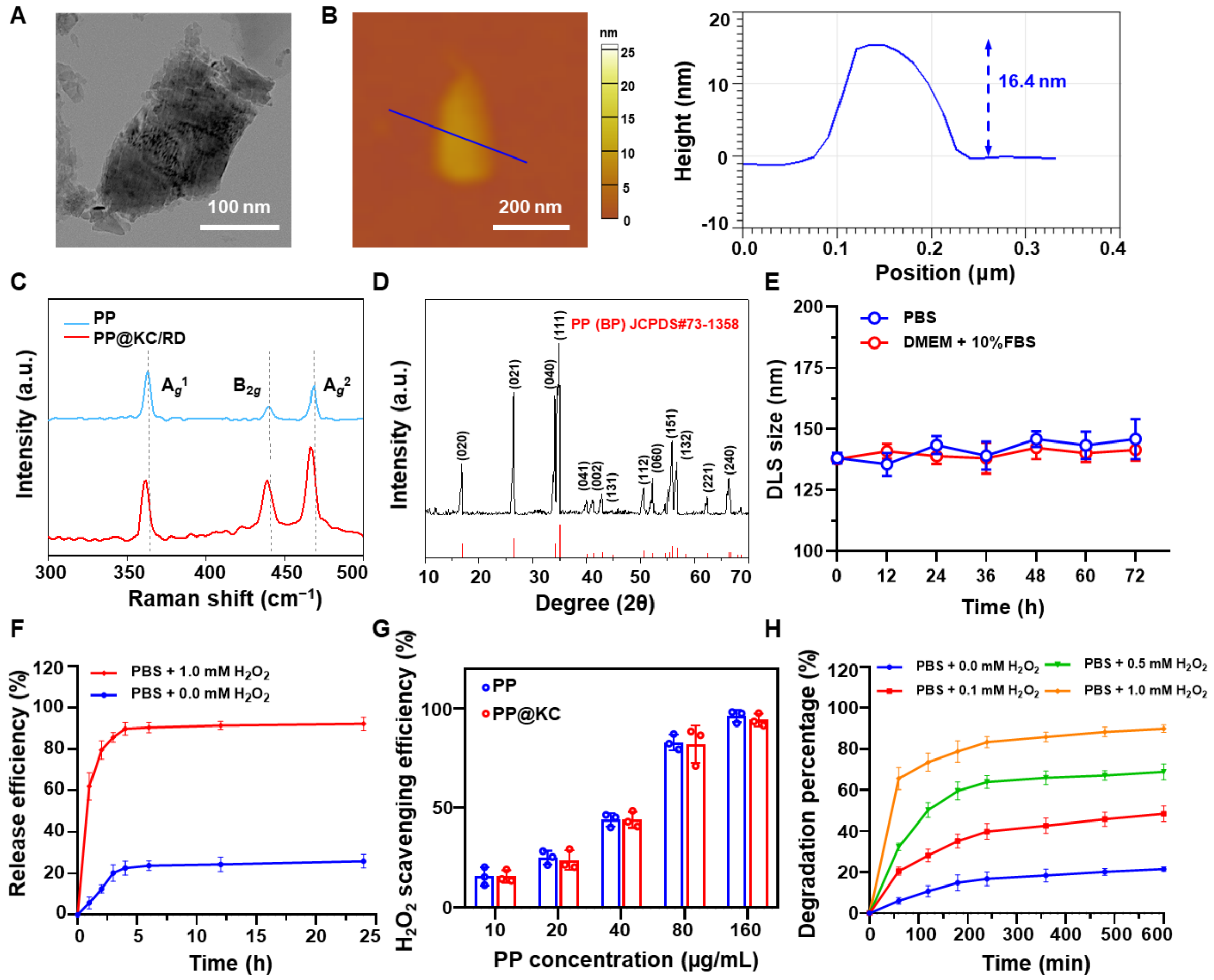

Figure S2 Characterization of PP@KC/RD and RD release studies. (A) TEM image, and (B) AFM image and the corresponding thickness profile of PP@KC/RD. Scale bar=100 nm. (C) Raman scattering spectra of PP@KC/RD. (D) The X-ray diffractometry (XRD) pattern of PP@KC/RD. (E) Time-course DLS (dynamic light scattering) sizes change of PP@KC/RD in PBS and DMEM containing 10% fetal bovine serum (*n* = 3). (F) Time-dependent RD release profiles from PP@KC/RD. The release was investigated using the dialysis method in PBS containing different concentrations of H_2O_2 (*n* = 3). (G) H_2O_2 scavenging efficiency of the nanosheets with various concentrations of PP and PP@KC (*n* = 3). (H) Degradation rate of PP@KC in PBS containing various concentrations of H_2O_2 (0, 0.1, 0.5, or 1.0 mmol/L) (*n* = 3). Data were shown as mean ± SD

Results and Discussion of **Figure S2**:

We investigated the ROS-responsive release of RD from PP@KC/RD, considering that atherosclerotic plaque environments contain high levels of reactive oxygen species (ROS) and PP are known to scavenge ROS species such as H_2O_2, leading to PP degradation[1–3]. Figure S2F demonstrates that PP@KC/RD exhibited a significant drug release efficiency of approximately

90% when exposed to 1.0 mmol/L H_2O_2, whereas only around 20% of the drug was released in the absence of H_2O_2 activation. Importantly, the capability of PP to ROS remained unaffected after surface coating with KC11-PEG-NH_2 (Figure S2G). Furthermore, to investigate the ROS-responsive degradation of PP@KC, we measured the absorbance of PP@KC in PBS solutions with different H_2O_2 concentrations (0, 0.1, 0.5, and 1.0 mmol/L)[1,4]. As shown in Figure S2H, the degradation of PP@KC was found to be H_2O_2-dependent, with the highest degradation (approximately 90%) observed in the presence of 1.0 mmol/L H_2O_2. Collectively, these findings demonstrate that the ROS-responsiveness of PP@KC not only minimizes RD premature release in the normal physiological microenvironment, but also enhances the drug concentration with a high level in atherosclerotic plaques, thereby enhancing therapeutic efficacy for atherosclerosis while reducing adverse effects.

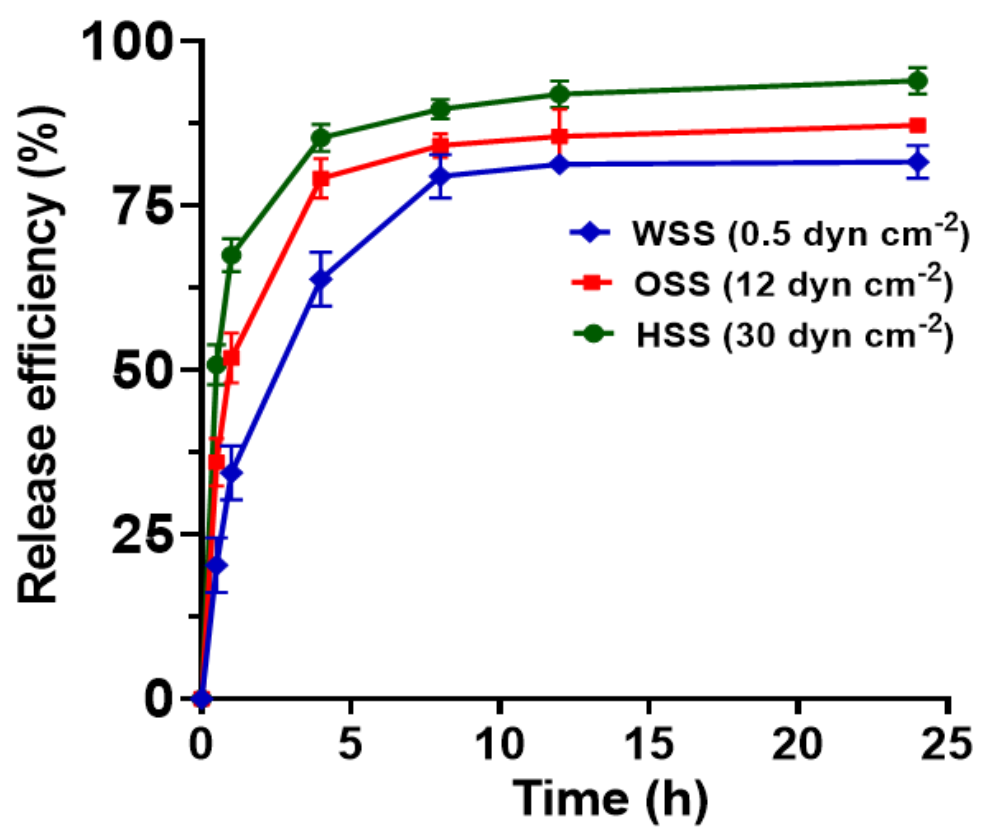

Figure S3 Parallel-plate flow-chamber evaluation of shear-dependent RD release from PP@KC/RD. Nanocarriers were immobilized on a collagen-IV-coated glass surface and exposed to oscillatory shear stress (OSS, 0.5 dyn cm^{-2}), low laminar shear stress (LSS, 12 dyn cm^{-2}), or high shear stress (HSS, 30 dyn cm^{-2}) for 24 h. Cumulative release profiles of RD under WSS, OSS, and HSS conditions were determined. Data are expressed as mean ± SD (n = 3).

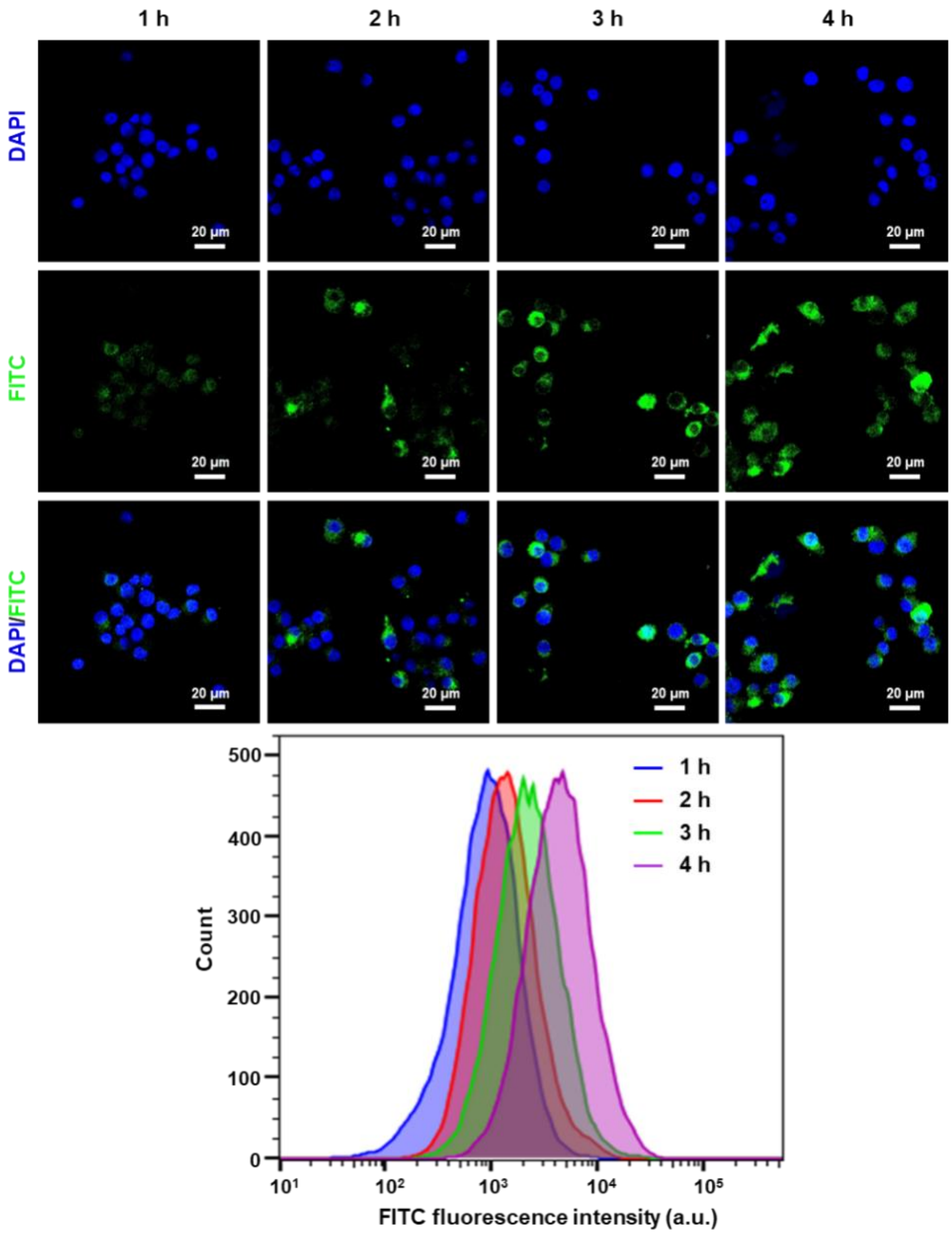

Figure S4 Cellular uptake analysis of PP@KC/FITC in LPS-stimulated RAW264.7 cells. CLSM images and flow cytometry histograms of RAW264.7 macrophages treated with PP@KC/FITC (50 μg/mL) for different time point (1, 2, 3, or 4 h). Scale bar=20 μm. The RAW264.7 macrophages pretreated with LPS (500 ng/mL) for 12 h. The cell nuclei were stained with DAPI (blue). The merge images are also presented in Fig. 2I.

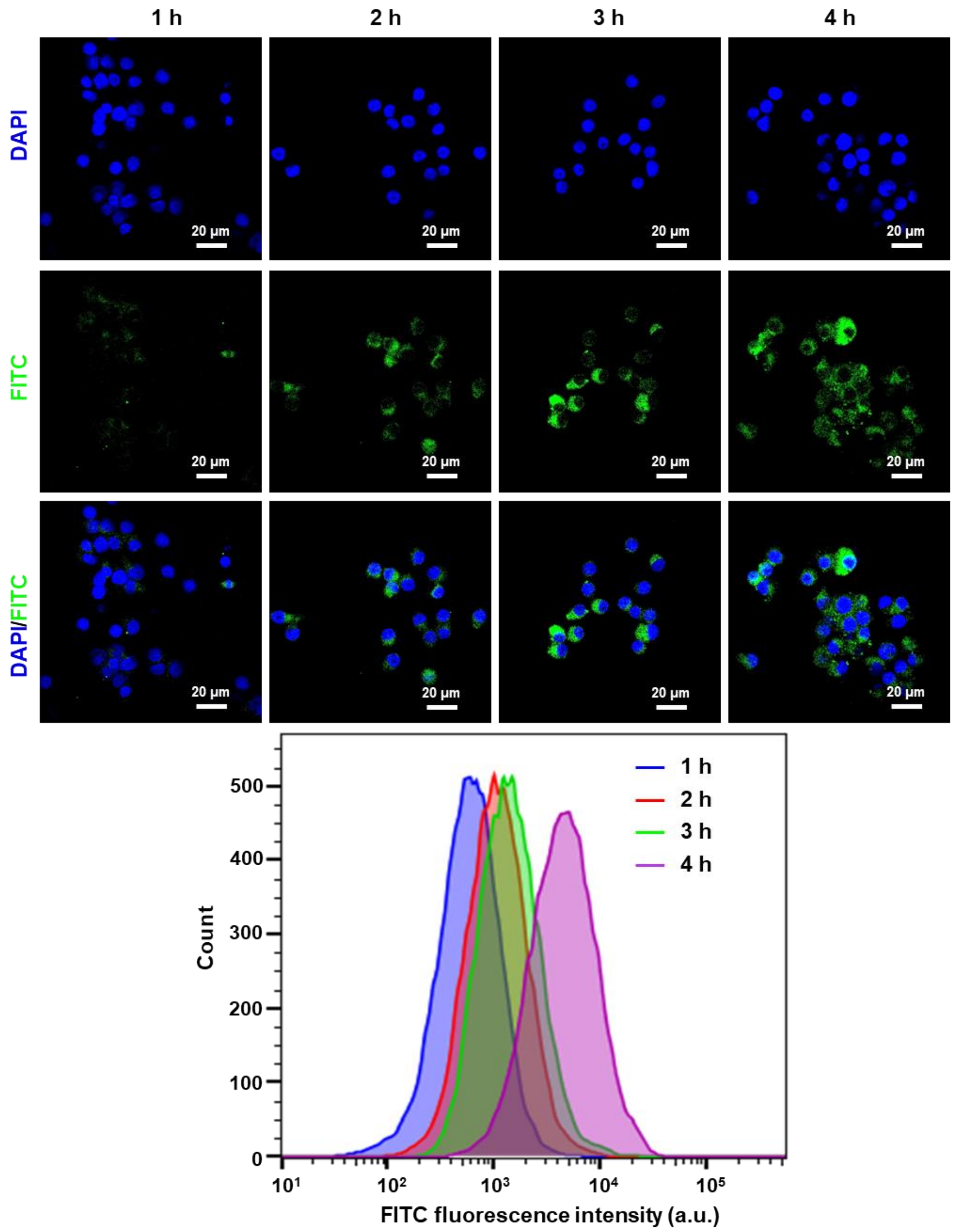

Figure S5 Cellular uptake analysis of PP@KC/FITC in ox-LDL-stimulated RAW264.7 cells. CLSM images and flow cytometry histograms of RAW264.7 macrophages treated with PP@KC/FITC (50 μg/mL) for different time point (1, 2, 3, or 4 h). Scale bar=20 μm.The RAW264.7 macrophages pretreated with 20 μg/mL ox-LDL for 12 h, and stained with DAPI (blue) to label their cell nuclei. The merge images are also presented in Fig. 2I.

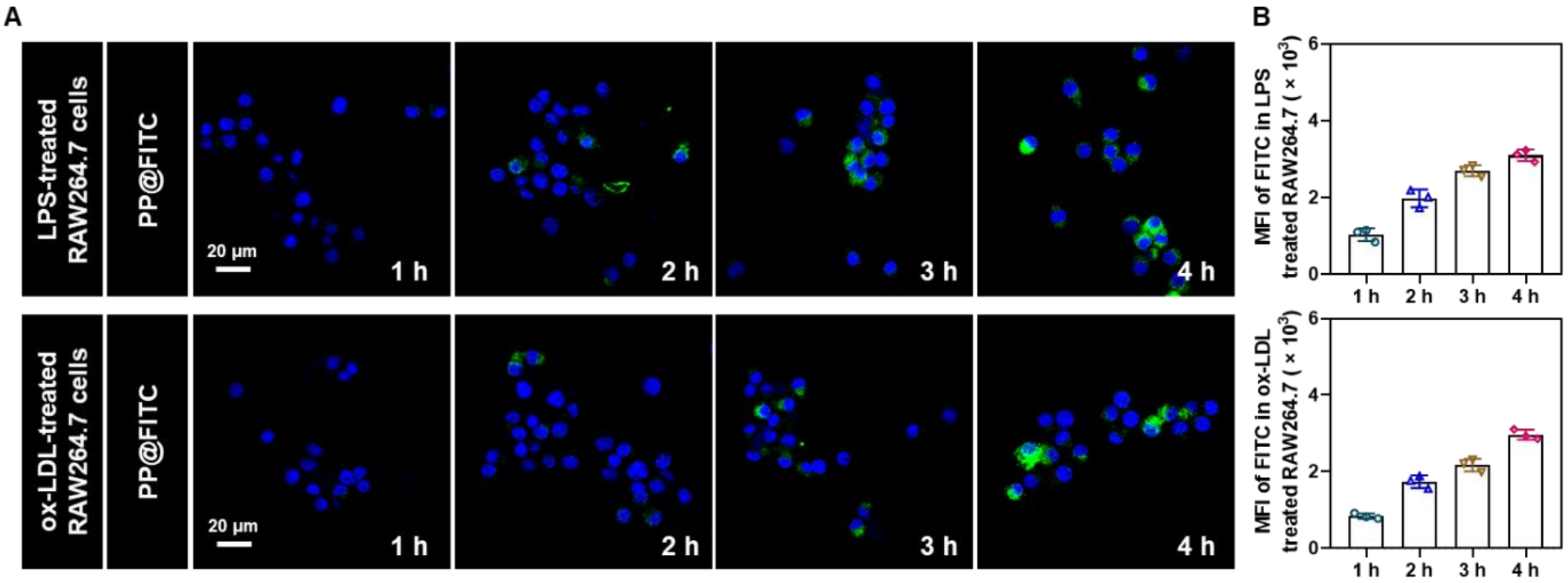

Figure S6 Cellular uptake analysis of PP@FITC in LPS or ox-LDL-stimulated RAW264.7 cells. (A) Time-dependent cellular uptake of PP@FITC in RAW264.7 cells detected by fluorescence microscope images. Blue: cell nuclei staining with DAPI. Scale bar=20 μm. (B) Quantitation of mean fluorescence intensity (MFI) of PP@FITC in RAW264.7 by flow cytometry analysis (n = 3). The LPS-treated RAW264.7 macrophages pretreated with 500 ng/mL LPS for 12 h; The ox-LDL-treated RAW264.7 macrophages pretreated with 20 μg/mL ox-LDL for 12 h. Data are shown as mean ± SD.

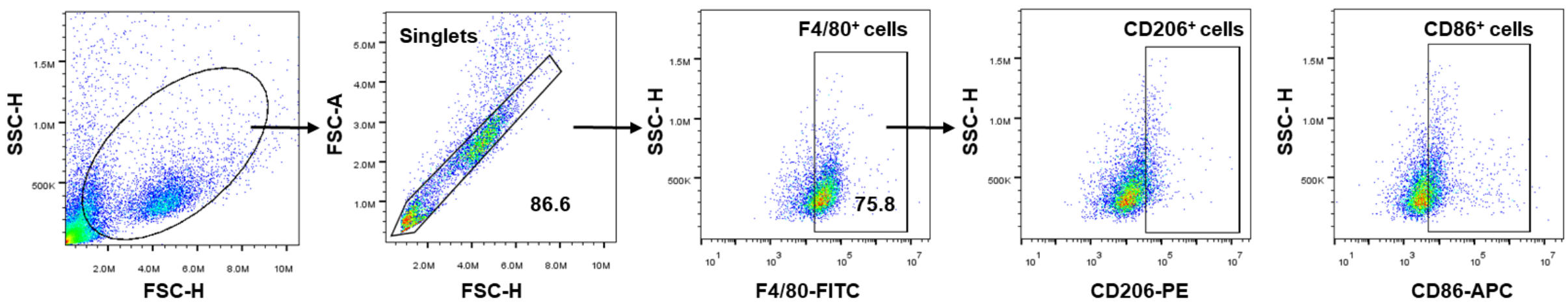

Figure S7 Gating strategy used in the flow cytometry evaluation of M2-like macrophage polarization. The RAW264.7 macrophages were preincubated with various formulations, including RD (10 nmol/L), PP@KC (50 μg/mL), and PP@KC/RD (50 μg PP@KC/RD/mL; 10 nmol/L RD) for 24 h, and treated with CD16/32 FC Block. The treated cells were then incubated with antibodies, including FITC rat anti-mouse F4/80 and APC rat anti-mouse CD86, and PE anti-mouse CD206 (MMR) antibody at 4 °C for 30 min. Cells were then gated on single cell populations (FSC-H/FSC-A), F4/80$^+$ cells (macrophage), CD206$^+$ cells (M2 macrophage) or CD86$^+$ cells (M1 macrophage).

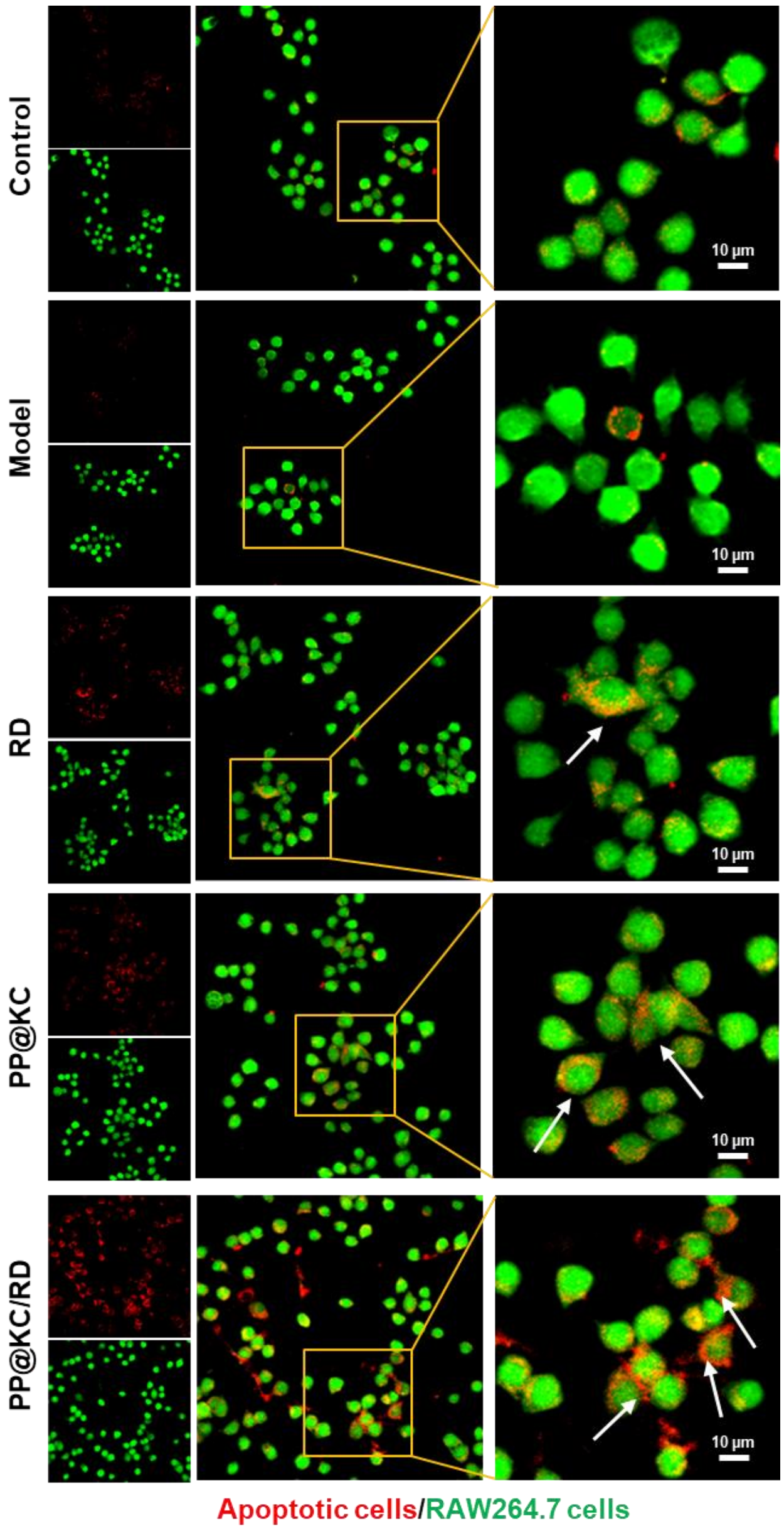

Figure S8 Investigation of RAW264.7 cells efferocytosis efficiency. Representative fluorescence microscope images of apoptotic cells internalization by macrophages. (Red: apoptotic cells; Green: RAW264.7 macrophages). Scale bar=10 μm. The RAW264.7 macrophages were preincubated with various formulations, including RD (10 nmol/L), PP@KC (50 μg/mL), and PP@KC/RD (50 μg PP@KC/RD/mL; 10 nmol/L RD) for 2 h, and labeled by CellTracker Green (1.5 μmol/L; Life Technologies) for 30 min, followed by co-incubated with CellTracker Red (2 μmol/L; Life Technologies)–labeled apoptotic cells at a 1:3 ratio for 2 h. The cells without any treatment and with LPS-only treatment served as the control and negative control (model), respectively.

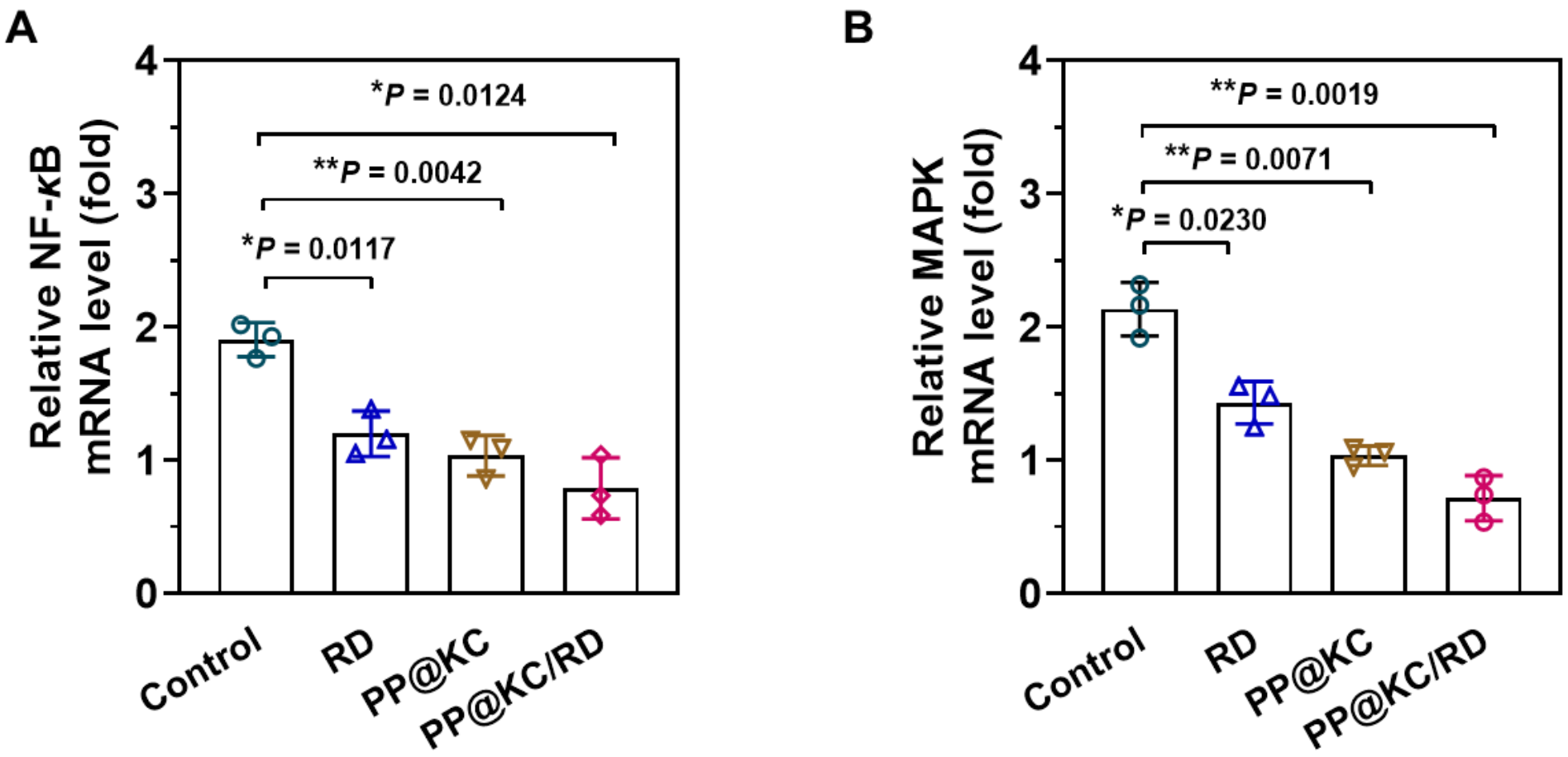

Figure S9 Expression of macrophage inflammation-resolving pathway genes after PP@KC/RD treatment. Relative mRNA expression of inflammatory-resolution genes (A) NF-*κ*B and (B) MAPK, measured by RT-qPCR in LPS/IFN-*γ* stimulated RAW264.7 cells treated with various formulations. RAW264.7 cells were stimulated with LPS/IFN-*γ* for 12 h, followed by co-incubation with either fresh medium (control) or the indicated treatments for 48 h. Data were analyzed using one-way ANOVA with Dunnett T3 *post hoc* test and shown as mean ± SD ($n = 3$ biologically independent samples). Statistical significance is indicated as *$P < 0.05$, **$P < 0.01$, and $P > 0.05$ indicate no significance.

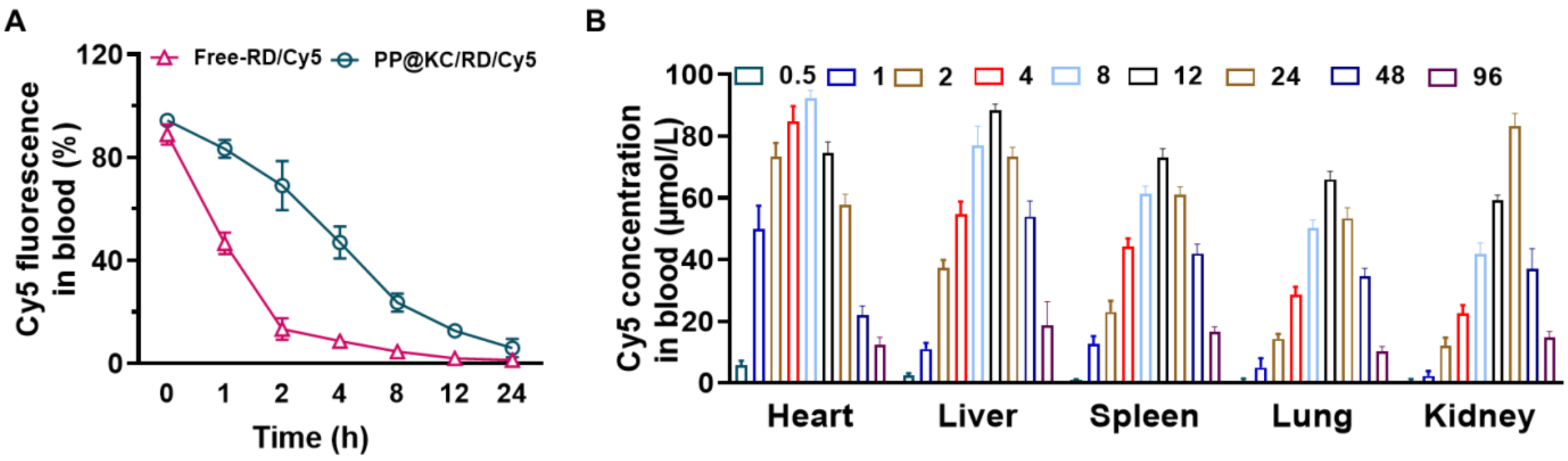

Figure S10 Pharmacokinetics and biodistribution of Cy5-labeled PP@KC/RD *in vivo*. (A) Plasma concentration-time profiles of Cy5 fluorescence following intravenous injection of Cy5-labeled nanomedicine versus free RD/Cy5 in mice. $n = 3$ per time point. (B) Biodistribution of Cy5-labeled PP@KC/RD in major organs at various time points (0.5, 1, 2, 4, 8, 12, 24, 48, and 96 h) post-injection, quantified by *ex vivo* fluorescence imaging. Data are expressed as mean Cy5 concentration (μmol/L) ± SD $n = 3$ per time point. Data are presented as mean ± SD.

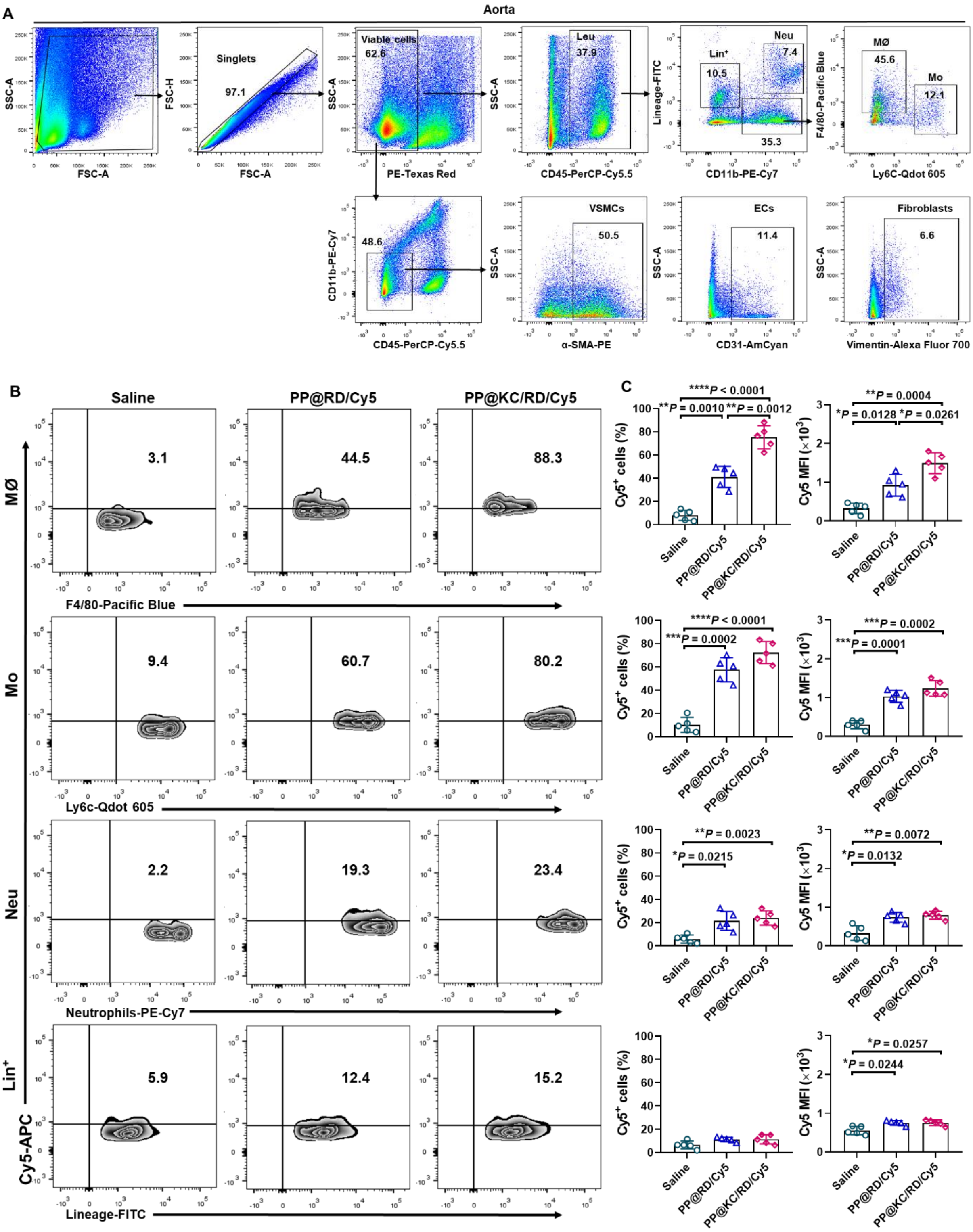
A
Aorta
Singlets
97.1
Viable cells
62.6
Leu
37.9
Lin+
10.5
Neu
7.4
35.3
MØ
45.6
Mo
12.1
48.6
VSMCs
50.5
ECs
11.4
Fibroblasts
6.6
SSC-A
FSC-A
FSC-H
PE-Texas Red
CD45-PerCP-Cy5.5
Lineage-FITC
CD11b-PE-Cy7
F4/80-Pacific Blue
Ly6C-Qdot 605
α-SMA-PE
CD31-AmCyan
Vimentin-Alexa Fluor 700
B
Saline
PP@RD/Cy5
PP@KC/RD/Cy5
MØ
3.1
44.5
88.3
F4/80-Pacific Blue
Mo
9.4
60.7
80.2
Ly6c-Qdot 605
Neu
2.2
19.3
23.4
Neutrophils-PE-Cy7
Lin+
5.9
12.4
15.2
Cy5-APC
Lineage-FITC
C
****P < 0.0001
**P = 0.0010
**P = 0.0012
**P = 0.0004
*P = 0.0128
*P = 0.0261
***P = 0.0002
***P = 0.0001
**P = 0.0023
*P = 0.0215
**P = 0.0072
*P = 0.0132
*P = 0.0257
*P = 0.0244
Cy5+ cells (%)
Cy5 MFI (×10³)
Saline
PP@RD/Cy5
PP@KC/RD/Cy5

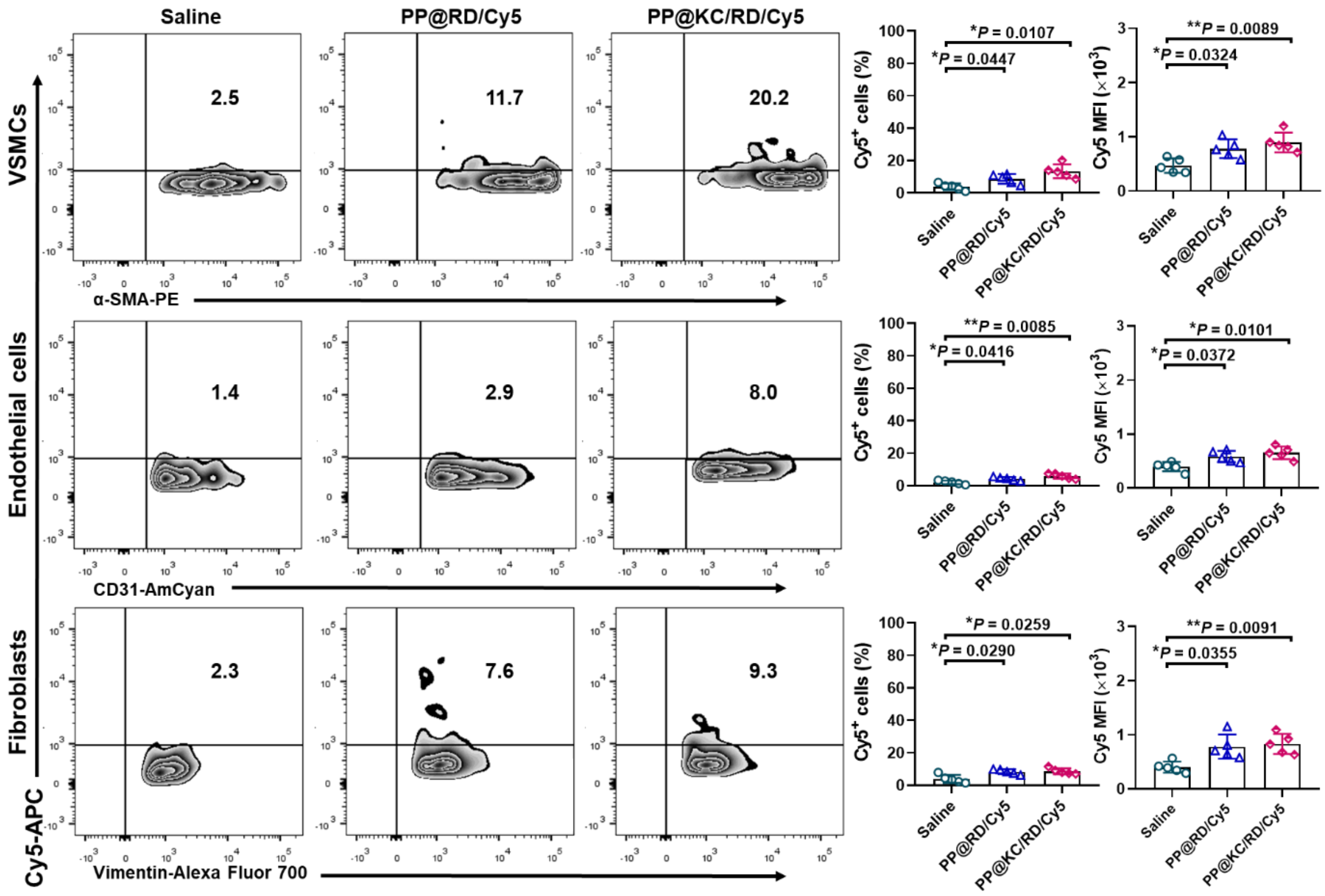

Figure S11 Vascular cell specificity of PP@KC/RD/Cy5 in atherosclerotic mice by flow cytometry analysis. (A) Gating strategy used in the flow cytometry evaluation of vascular cell specificity of targeted PP@KC/RD/Cy5 and non-targeted PP@RD/Cy5 in plaque-bearing *ApoE*$^{-/-}$ mice. Macrophages (M*ø*) were identified as CD45^{hi}, CD11b^{hi}, Lin^{low}, and F4/80^{hi} cells (with Lin^{hi} defined as CD45R^{+}, CD90.2^{+}, CD49b^{+}, Ter119^{+}, Ly-6G^{+}, and NK1.1^{+}). Neutrophils were identified as CD45^{hi}, CD11b^{hi}, and Lin^{hi} cells. Ly-6C^{hi} monocytes (Mo) were identified as CD45^{hi}, CD11b^{hi}, Lin^{low}, and Ly-6C^{hi} cells. Vascular smooth muscle cells (VSMCs) were defined as CD45^{hi}, CD11b^{low}, and *α*-SMA^{hi} cells. Endothelial cells were defined as CD45^{hi}, CD11b^{low}, and CD31^{hi} cells. Fibroblasts were defined as CD45^{hi}, CD11b^{low}, and Vimentin^{hi} cells. (B) Representative flow cytometry plots of PP@KC/RD/Cy5 and PP@RD/Cy5 uptake by the seven vascular cells. (C) Cellular uptake efficiency of PP@KC/RD/Cy5 and PP@RD/Cy5 and MFI of the aortic cells. All data are expressed as mean ± SD (n = 5). Statistical significance was analyzed using one-way ANOVA with a Games-Howell *post hoc* test. *P< 0.05, **P< 0.01, ***P< 0.001 and ****P< 0.0001.

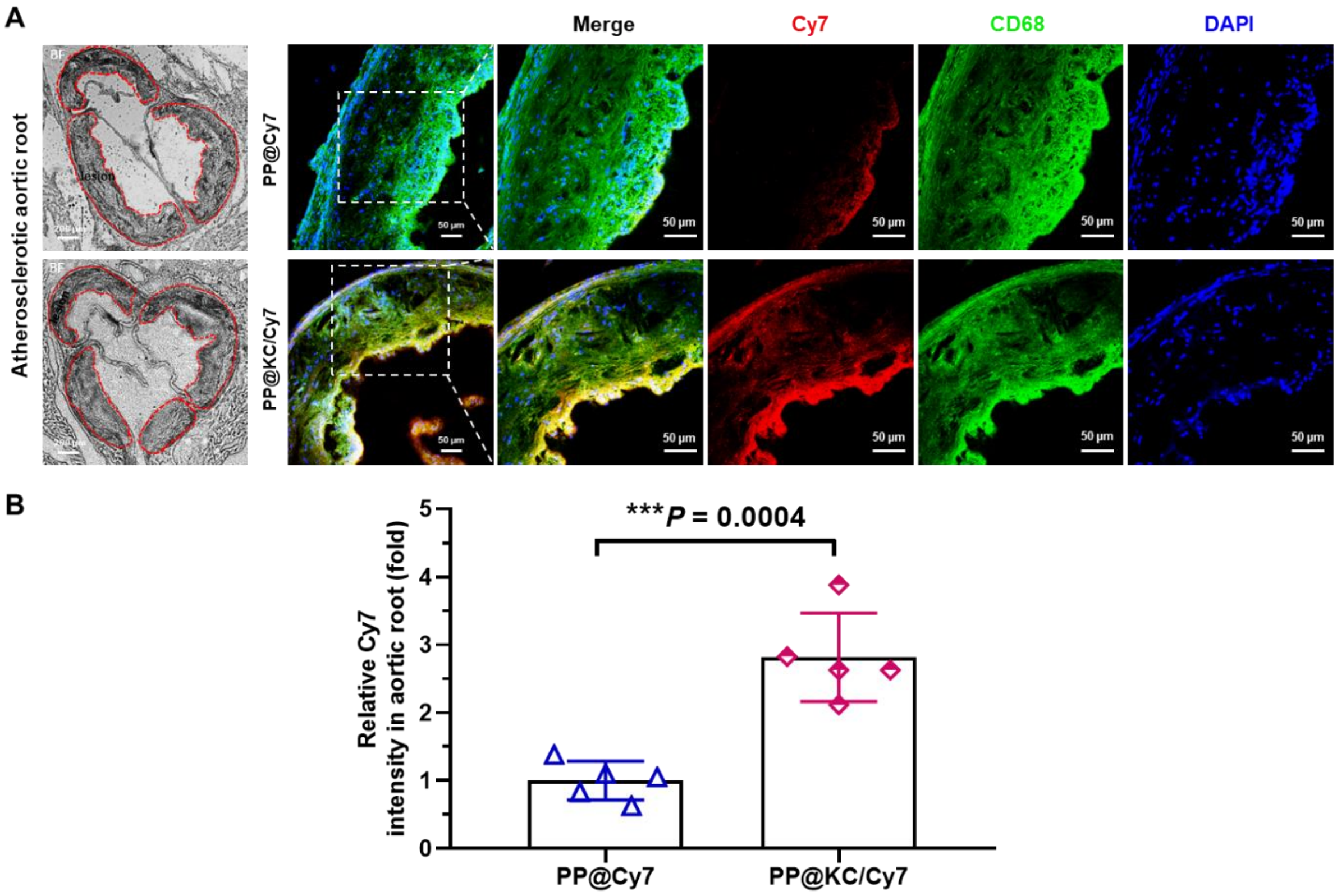

Figure S12 Improved accumulation of PP@KC/Cy7 in aortic roots. (A) CLSM images of PP@KC/Cy7 accumulation in lesional macrophages of the aortic roots. Red: PP@KC/Cy7 or PP@Cy7; Green: macrophage maker was stained with CD68 antibody; Blue: cell nuclei were stained with DAPI. Scale bar=50 or 200 μm. (B) Quantification of the fluorescence intensity ratio (*versus* PP@Cy7) of Cy7 in the aortic roots sections 12 h after the administration of PP@Cy7 or PP@KC/Cy7 in atherosclerotic *ApoE*$^{-/-}$ mice. Data are shown as mean ± SD (n = 5), and were analyzed using unpaired two-tailed Student's t-test. ***P< 0.001.

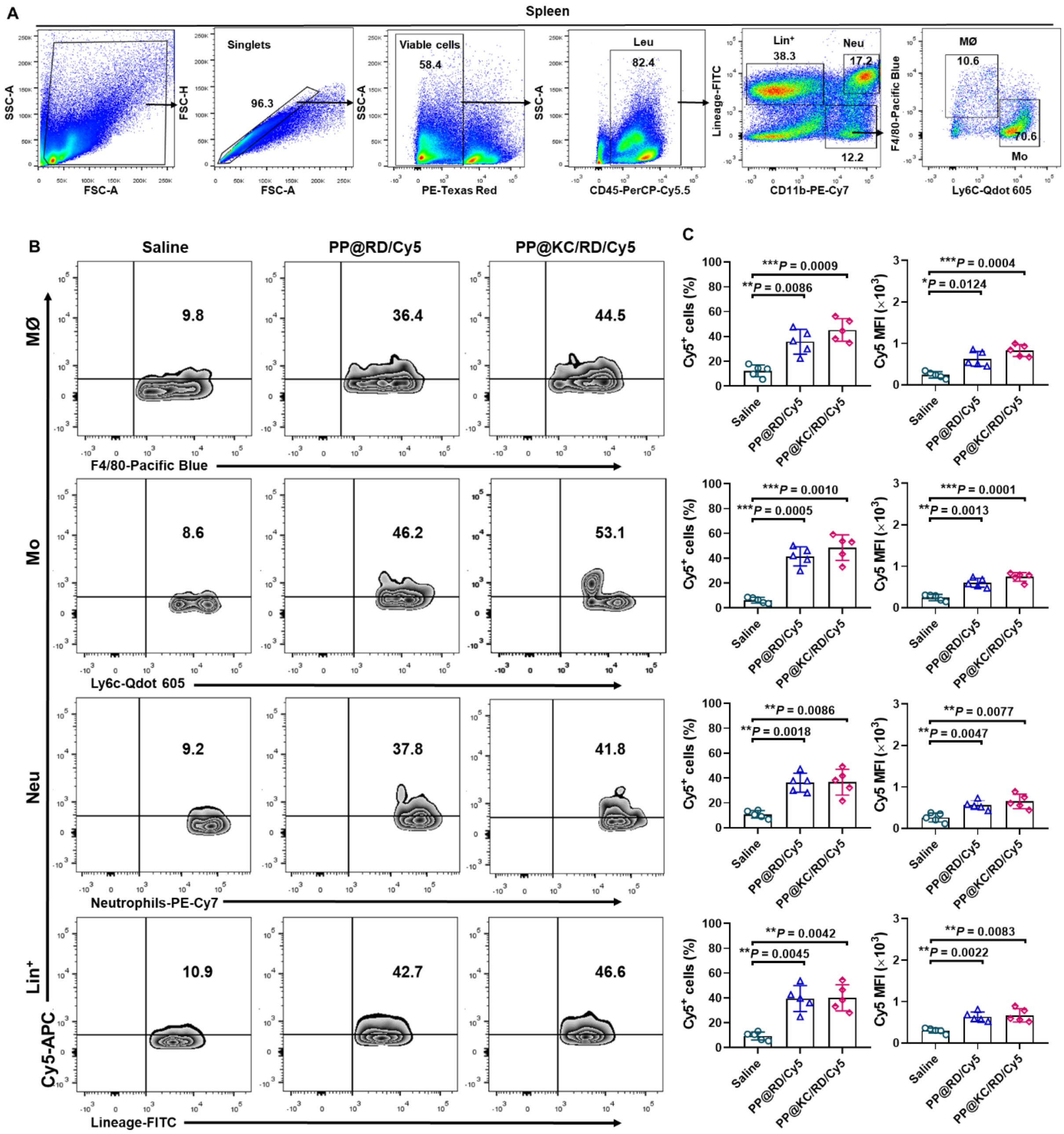

Figure S13 Splenic cell specificity of PP@KC/RD/Cy5 in atherosclerotic mice by flow cytometry analysis. (A) Gating strategy used in the flow cytometry evaluation of splenic cell specificity of targeted PP@KC/RD/Cy5 and non-targeted PP@RD/Cy5 in plaque-bearing *ApoE*$^{-/-}$ mice. Macrophages (M*ø*) were identified as CD45^{hi}, CD11b^{hi}, Lin^{low}, and F4/80^{hi} cells (with Lin^{hi} defined as CD45R^{+}, CD90.2^{+}, CD49b^{+}, Ter119^{+}, Ly-6G^{+}, and NK1.1^{+}). Neutrophils were identified as CD45^{hi}, CD11b^{hi}, and Lin^{hi} cells. Ly-6C^{hi} monocytes (Mo) were identified as CD45^{hi}, CD11b^{hi}, Lin^{low}, and Ly-6C^{hi} cells. (B) Representative flow cytometry plots of PP@KC/RD/Cy5 and PP@RD/Cy5 uptake by macrophages, Ly-6C^{hi} monocytes, neutrophils, and Lin^{+} CD11^{-} lymphocytes. (C) Cellular uptake efficiency of PP@KC/RD/Cy5 and PP@RD/Cy5 and MFI of the spleen cells. All data are expressed as mean ± SD (n = 5). Statistical

significance was analyzed using one-way ANOVA with a Games-Howell *post hoc* test. *$P< 0.05$, **$P< 0.01$, and ***$P< 0.001$.

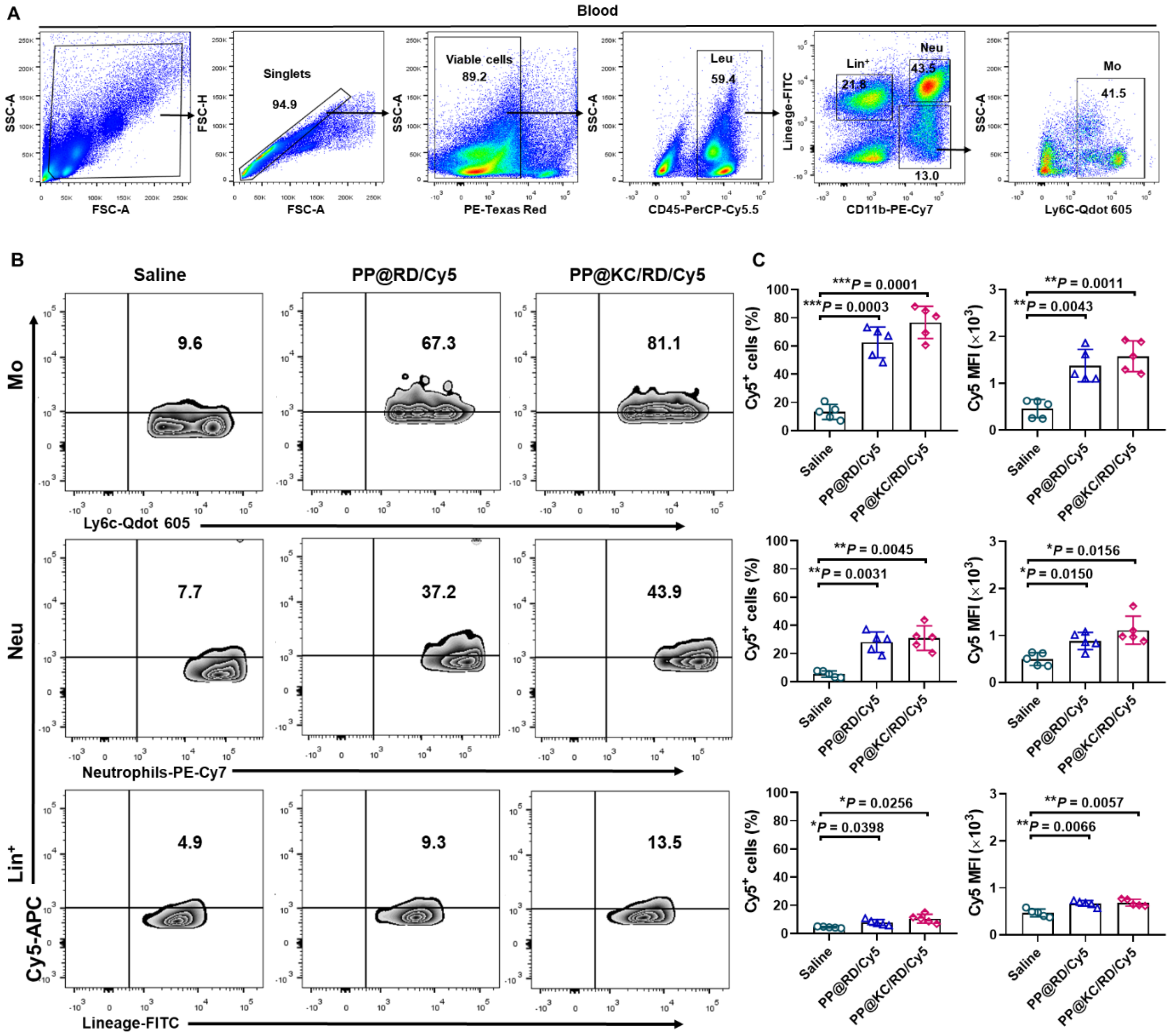

Figure S14 Blood cell specificity of PP@KC/RD/Cy5 in atherosclerotic mice by flow cytometry analysis. (A) Gating strategy used in the flow cytometry evaluation blood cell specificity of targeted PP@KC/RD/Cy5 and non-targeted PP@RD/Cy5 in plaque-bearing *ApoE*$^{-/-}$ mice. Ly-6C^{hi} monocytes (Mo) were identified as CD45^{hi}, CD11b^{hi}, Lin^{low}, and Ly-6C^{hi} cells. Neutrophils were identified as CD45^{+}, CD11b^{hi}, and Lin^{hi} cells. Lin^{hi} cells were defined as CD45R^{+}, CD90.2^{+}, CD49b^{+}, Ter119^{+}, Ly-6G^{+}, and NK1.1^{+} cells. (B) Representative flow cytometry plots of PP@KC/RD/Cy5 and PP@RD/Cy5 uptake by Ly-6C^{hi} monocytes, neutrophils, and Lin^{+} CD11b^{-} cells. (C) Cellular uptake efficiency of PP@KC/RD/Cy5 and PP@RD/Cy5 and MFI of the blood cells. All data are expressed as mean ± SD (n = 5). Statistical significance was analyzed using one-way ANOVA with a Games-Howell *post hoc* test. *$P< 0.05$, **$P< 0.01$, and ***$P< 0.001$.

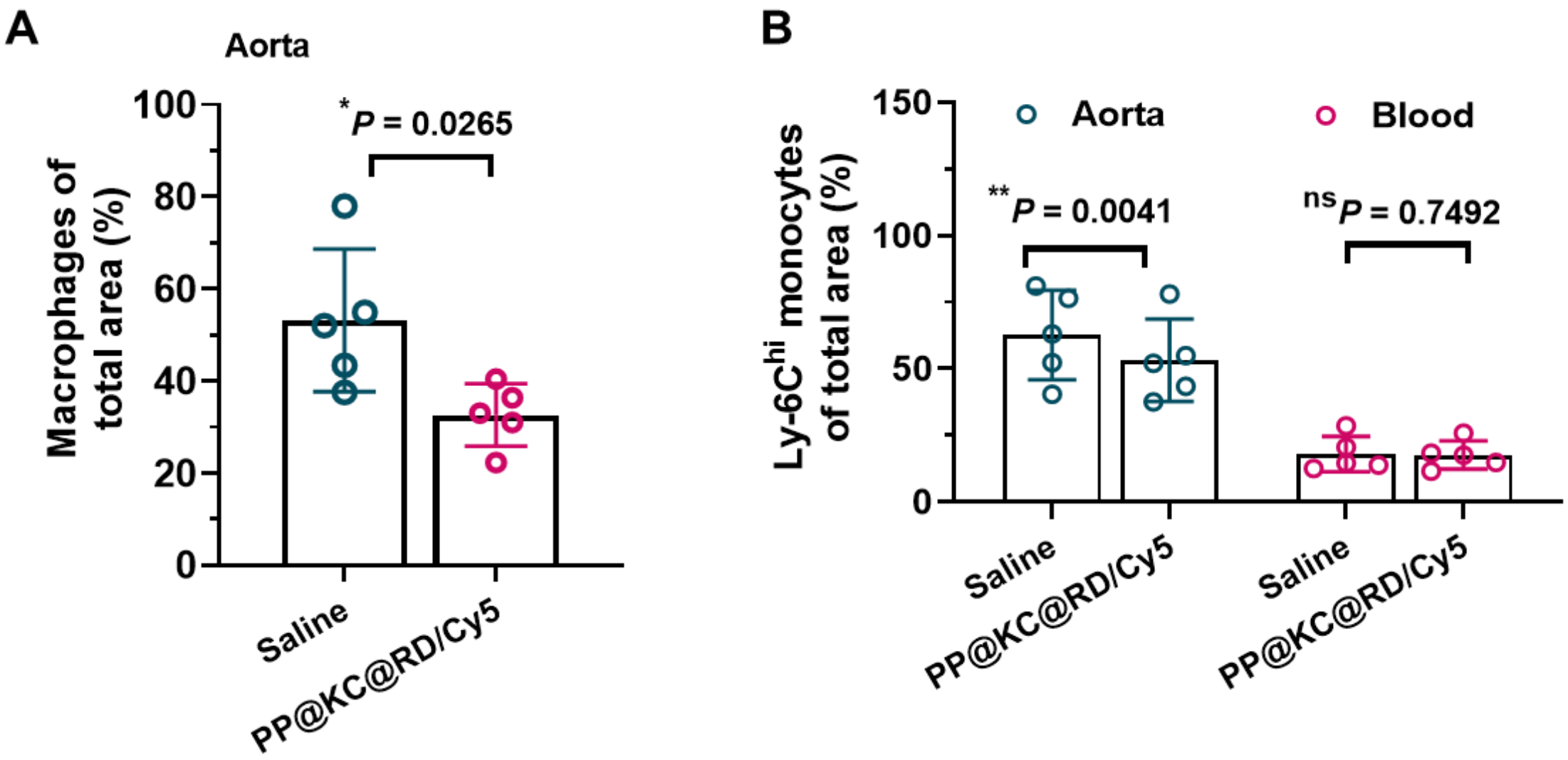

Figure S15 PP@KC/RD/Cy5 does not alter macrophage or monocyte abundance after prolonged treatment. (A) Absolute counts of aortic $CD45^{hi}CD11b^{hi}F4/80^{hi}$ macrophages after 12 weeks of saline or PP@KC/RD/Cy5 treatment. (B) Frequencies of Ly-$6C^{hi}$ monocytes in the aorta, and blood. Data are presented as mean ± SD (*n* = 5 mice per group). n.s. indicates not significant by unpaired two-tailed Student's *t*-test.

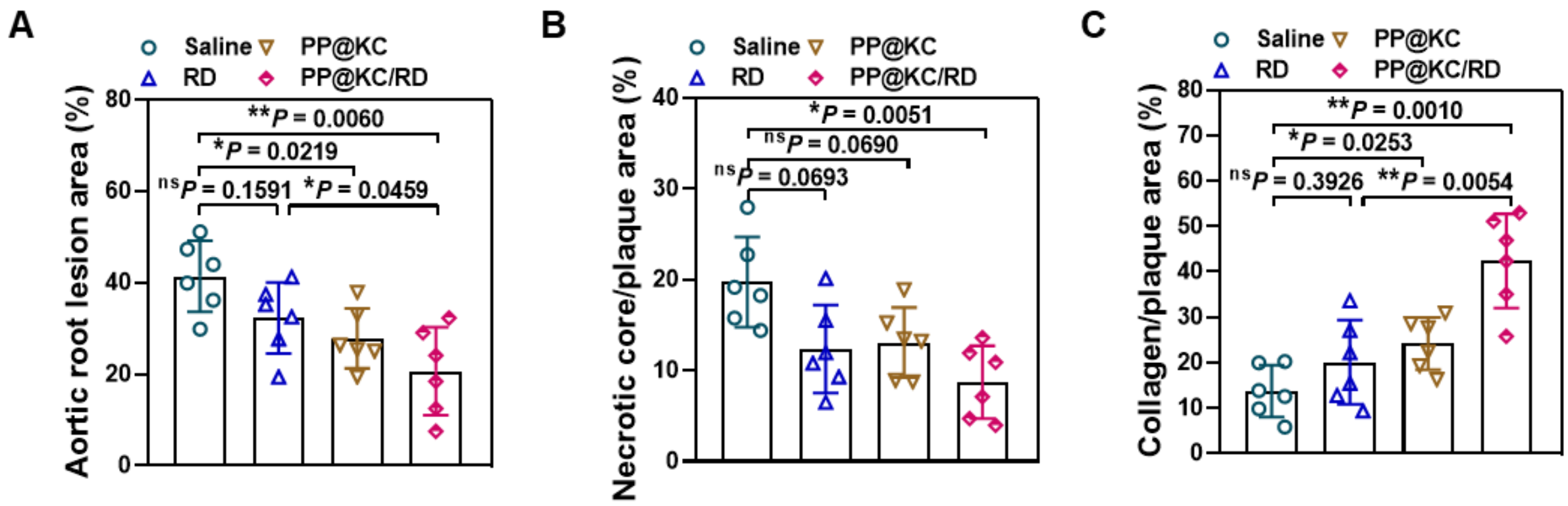

Figure S16 PP@KC/RD therapy reduced lesion areas and improved plaque stability. Quantitation of the ratio of (A) ORO-positive area, (B) necrotic area, and (C) collagen area to the plaque area of the aortic root sections (*n* = 6). Atherosclerotic *ApoE*$^{-/-}$ mice were sacrificed after 12-week biweekly treatments, including saline, RD (100 ng of RD/mouse), PP@KC (10 µg of PP@KC/mouse), PP@KC/RD (10 µg of PP@KC containing 100 ng of RD/mouse). Data are shown as mean ± SD and analysed using one-way ANOVA with a Dunnett T3 *post hoc* test. *$P<$ 0.05, **$P<$ 0.01, and $P>$ 0.05 denotes no significance.

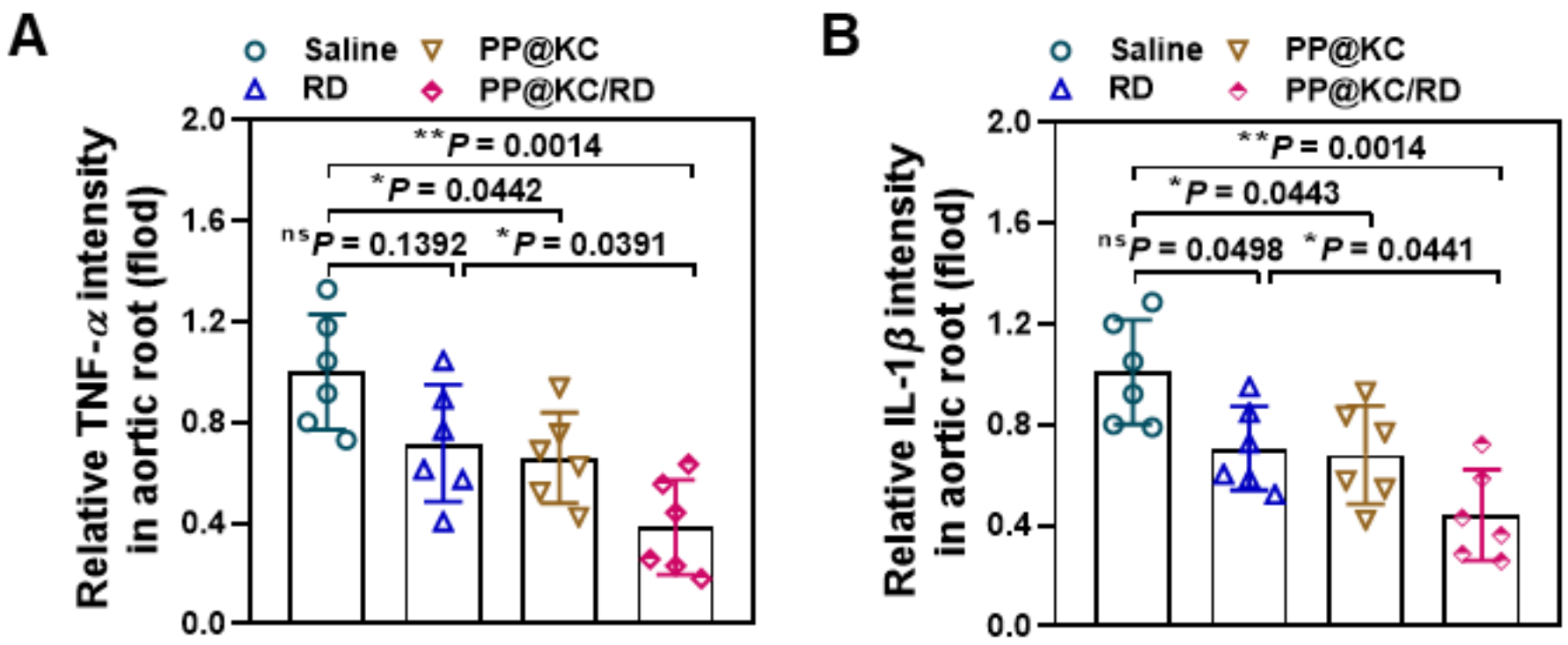

Figure S17 PP@KC/RD therapy decreased lesional TNF-*α* and IL-1*β* expression. Quantitative analysis of the ratio (*versus* saline) of (A) TNF-*α*, and (B) IL-1*β* in aortic root sections after different treatments. including saline, RD (100 ng of RD/mouse), PP@KC (10 μg of PP@KC/mouse), PP@KC/RD (10 μg of PP@KC containing 100 ng of RD/mouse). All data are shown as mean ± SD (n = 6), and analysed using one-way ANOVA with a Dunnett T3 *post hoc* test. *P< 0.05, **P< 0.01, and P> 0.05 denotes no significance.

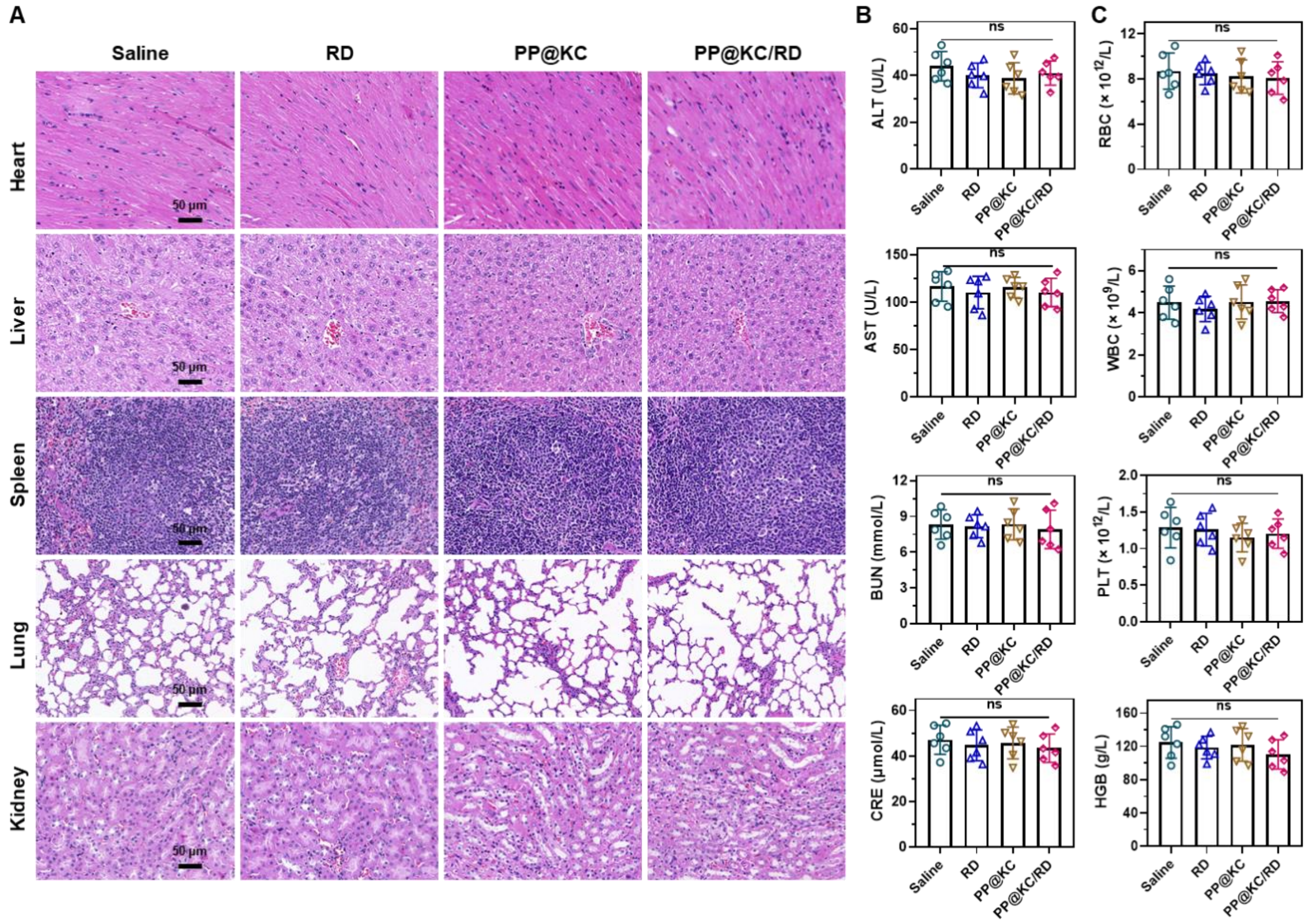

Figure S18 Safety evaluation of plaque-bearing *ApoE*$^{-/-}$ mice after 12 weeks of treatment with various formulations. The detailed various treatments are as follows: saline, RD, PP@KC, PP@KC/RD. (A) H&E staining images of major organs collected from *ApoE*$^{-/-}$ mice after different treatments. Scale bar=50 μm. (B) Serum levels of aspartate aminotransferase (AST), alanine aminotransferase (ALT), blood urea nitrogen (BUN), and creatinine (CRE). (C) Typical hematological parameters of white blood cell (WBC), red blood cell (RBC), hemoglobin (HGB), and platelet (PLT). All data are expressed as mean ± SD (n = 6). Statistical significance was analyzed using one-way ANOVA with a Tukey *post hoc* test.

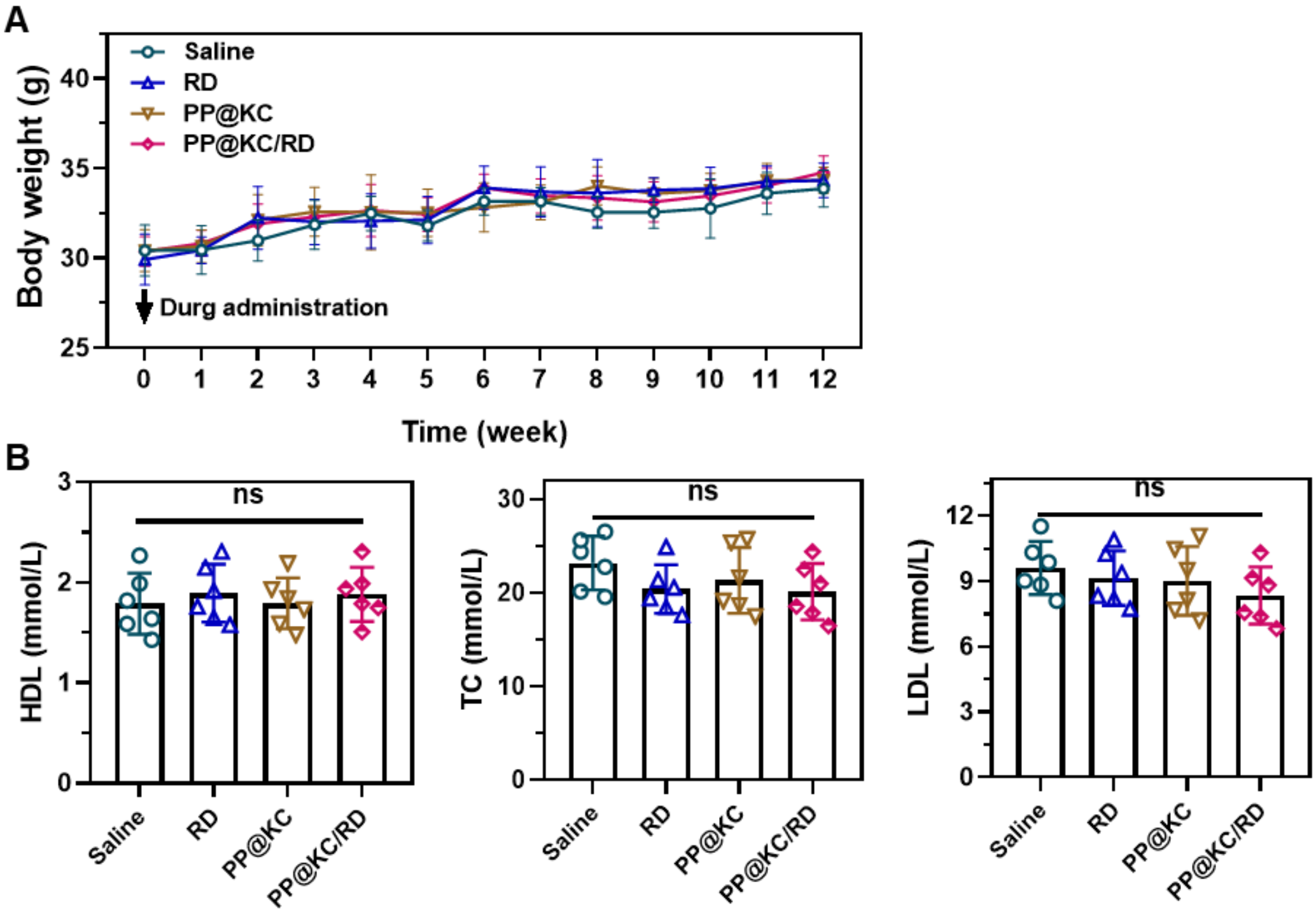

Figure S19 Analysis of body weight and blood lipid of atherosclerotic *ApoE*$^{-/-}$ mice after receaving various treatments for 12 weeks. The detailed various treatments are as follows: saline, RD, PP@KC, PP@KC/RD. (A) Body weight of atherosclerotic *ApoE*$^{-/-}$ mice treated with various formulations (n = 6). (B) Serum levels of high density lipoprotein (HDL), total cholesterol (TC), and low-density lipoprotein (LDL) (n = 6). No significant differences in the levels of serum HDL, TC, and LDL were observed, suggesting that the PP@KC/RD treatment did not affect the levels of cholesterol and blood glucose *in vivo*. All data are expressed as mean ± SD (n = 6). Statistical significance was analyzed using one-way ANOVA with a Tukey *post hoc* test.

Table S1 Antibodies used for vascular cell specificity analysis in Fig. S11.

Antibody	Cat#	Concentration for use
PerCP/Cyanine5.5 anti-mouse CD45	BD Biosciences; 550994	3.0 μg/mL
FITC anti-mouse CD90.2	BD Biosciences; 561973	1.5 μg/mL
FITC anti-mouse CD45R	BD Biosciences; 553088	2.5 μg/mL
FITC anti-mouse CD49b	BD Biosciences; 553857	2.5 μg/mL
FITC anti-mouse NK1.1	BD Biosciences; 553164	2.5 μg/mL
FITC anti-mouseTer119	BioLegend; 116206	2.5 μg/mL
FITC anti-mouse Ly6G	BioLegend; 127606	2.5 μg/mL
PE/Cyanine7 anti-mouse CD11b	BD Biosciences; 552850	3.0 μg/mL
BV421 anti-mouse F4/80	BD Biosciences; 565411	2.5 μg/mL
BV605 anti-mouse Ly-6C	BD Biosciences; 563011	2.5 μg/mL
BV510 anti-mouse CD31	BD Biosciences; 740124	2.5 μg/mL
PE anti-mouse alpha-Smooth muscle actin	Novus; NBP2-34522PE	1.5 μg/mL
Alexa Fluor® 700 anti-mouse Vimentin	R&D Systems; IC2105N	1.5 μg/mL

Table S2 Antibodies used for splenic cells specificity analysis in Figure S13.

Antibody	Cat#	Concentration for use
PerCP/Cyanine5.5 anti-mouse CD45	BD Biosciences; 550994	3.0 μg/mL
FITC anti-mouse CD90.2	BD Biosciences; 561973	1.5 μg/mL
FITC anti-mouse CD45R	BD Biosciences; 553088	2.5 μg/mL
FITC anti-mouse CD49b	BD Biosciences; 553857	2.5 μg/mL
FITC anti-mouse NK1.1	BD Biosciences; 553164	2.5 μg/mL
FITC anti-mouseTer119	BioLegend; 116206	2.5 μg/mL
FITC anti-mouse Ly6G	BioLegend; 127606	2.5 μg/mL
PE/Cyanine7 anti-mouse CD11b	BD Biosciences; 552850	3.0 μg/mL
BV421 anti-mouse F4/80	BD Biosciences; 565411	2.5 μg/mL
BV605 anti-mouse Ly-6C	BD Biosciences; 563011	2.5 μg/mL

Table S3 Antibodies used for blood cells specificity analysis in Figure S14.

Antibody	Cat#	Concentration for use
PerCP/Cyanine5.5 anti-mouse CD45	BD Biosciences; 550994	3.0 μg/mL
FITC anti-mouse CD90.2	BD Biosciences; 561973	1.5 μg/mL
FITC anti-mouse CD45R	BD Biosciences; 553088	2.5 μg/mL
FITC anti-mouse CD49b	BD Biosciences; 553857	2.5 μg/mL
FITC anti-mouse NK1.1	BD Biosciences; 553164	2.5 μg/mL
FITC anti-mouseTer119	BioLegend; 116206	2.5 μg/mL
FITC anti-mouse Ly6G	BioLegend; 127606	2.5 μg/mL
PE/Cyanine7 anti-mouse CD11b	BD Biosciences; 552850	3.0 μg/mL
BV605 anti-mouse Ly-6C	BD Biosciences; 563011	2.5 μg/mL

Table S4 Primer sequences used for the RT-qPCR analysis of polarization and efferocytosis of RAW264.7 cells.

Gene	Forward primer (5′–3′)	Reverse primer (5′–3′)
PPAR-γ	GCAGCTACTGCATGTGATCAAGA	GTCAGCGGGTGGACTTTC
MerTK	CAGGGCCTTTACCAGGGAGA	TGTGTGCTGGATGTGATCTTC
CaMKIIγ	TACAGTGAAGCTGATGCCAG	TTGACACCGCCATCTGACTT
MAPK	CACAGCACCTCAGCAATGAT	AGGCCTATCTTCCCAGGAAA
NF-κB	GACACGACAGAATCCTCAGCATCC	CCACCAGCAGCAGCAGACATG
ß-actin	GTCCCTCACCCTCCCAAAAG	GCTGCCTCAACACCTCAACCC

References

1. Gao C, Huang Q, Liu C, Kwong CHT, Yue L, Wan JB, et al. Treatment of atherosclerosis by macrophage-biomimetic nanoparticles *via* targeted pharmacotherapy and sequestration of proinflammatory cytokines. *Nat Commun* 2020;**11**:2622.
2. Cheng J, Zhang R, Li C, Tao H, Dou Y, Wang Y, et al. A targeting nanotherapy for abdominal aortic aneurysms. *J. Am. Coll. Cardiol* 2018;**72**:2591–605.
3. Hou J, Wang H, Ge Z, Zuo T, Chen Q, Liu X, et al. Treating acute kidney injurywith antioxidative black phosphorus nanosheets. *Nano. Lett* 2020;**20**:1447–54.
4. Liang X, Li H, Li X, Tian X, Zhang A, Luo Q, et al. Highly sensitive H_2O_2-scavenging nano-bionic system for precise treatment of atherosclerosis. *Acta Pharm. Sin. B* 2023;**13**:372–89.
5. Chen W, Schilperoort M, Cao Y, Shi J, Tabas I, Tao W. Macrophage-targeted nanomedicine for the diagnosis and treatment of atherosclerosis. *Nat Rev Cardiol* 2022;**19**: 228–49.
6. Flores A M, Hosseini-Nassab N, Jarr KU, Ye J, Zhu X, Wirka R, et al. Pro-efferocytic nanoparticles are specifically taken up by lesional macrophages and prevent atherosclerosis. *Nat Nanotechnol* 2020;**15**:154–61.
7. Doran AC, Ozcan L, Cai B, Zheng Z, Fredman G, Rymond CC, et al. CAMKIIγ suppresses an efferocytosis pathway in macrophages and promotes atherosclerotic plaque necrosis. *J Clin Invest* 2017;**127**:4075–89.
8. Fan X, Han J, Zhong L, Zheng W, Shao R, Zhang Y, et al. Macrophage-derived GSDMD plays an essential role in atherosclerosis and cross talk between macrophages *via* the mitochondria-STING-IRF3/NF-κB axis. *Arterioscler Thromb Vasc Biol* 2024;**44**:1365–78.
